# Effectiveness of a telephone-based randomised clinical trial targeting obesity risk of preschool-aged children: An extension study during the COVID-19 pandemic

**DOI:** 10.1038/s41366-025-01869-4

**Published:** 2025-08-14

**Authors:** Li Ming Wen, Huilan Xu, Zoe Chen, Alison Hayes, Philayrath Phongsavan, Sarah Taki, Erin Kerr, Danielle Jawad, Lisa Simone, Chris Rissel, Louise A. Baur

**Affiliations:** 1 Health Promotion Unit, Population Health Research & Evaluation Hub, Sydney Local Health District, Camperdown, NSW Australia; 2 Sydney School of Public Health, Faculty of Medicine and Health, The University of Sydney, Camperdown, NSW Australia; 3 NHMRC Centre of Research Excellence in the Early Prevention of Obesity in Childhood (EPOCH), Melbourne, VIC Australia; 4 The Charles Perkins Centre, The University of Sydney, Camperdown, NSW Australia; 5 Sydney Institute for Women, Children and Their Families, Sydney Local Health District, Camperdown, NSW Australia; 6 Sydney Medical School, The University of Sydney, Camperdown, NSW Australia; 7 College of Medicine and Public Health, Rural and Remote Health, SA and NT, Flinders University, Darwin, NT Australia; 8 Specialty of Child and Adolescent Health, Sydney Medical School, The University of Sydney, Camperdown, NSW Australia

**Keywords:** Lifestyle modification, Patient education, Paediatrics

## Abstract

**Background:**

There is a great need for determining the effectiveness of telephone-based early obesity interventions targeting preschool-aged children. This was particularly important during the COVID-19 pandemic when most face-to-face health promotion programs were suspended. The aim of this study was to determine the effects of a two-year telephone-based intervention on body mass index (BMI), eating habits, active play, and screen time behaviours among preschool-aged children.

**Methods:**

We conducted an extension study to a randomised controlled trial (RCT) with 662 mother-child dyads at ages 2–3 years in 2019–20 in the Greater Sydney metropolitan area of New South Wales (NSW), Australia. In 2020–22, we extended the RCT for another two years, with one-year intervention (3–4 years) and one-year follow-up (4–5 years). Participants remained in the same group allocation as the original trial. The intervention comprised five nurse-led telephone support calls and SMS plus mailed intervention booklets to mothers to promote the health behaviours of their children from ages 2 to 4 years. The primary outcome was children’s BMI, with weight and height measured at ages 3, 4, and 5 years. We conducted intention-to-treat analysis with a multiple imputation approach. Mixed linear models were built to compare the outcomes between intervention and control groups. Sub-group analysis by household income was also conducted.

**Results:**

Of the 662 mothers, 537 (81%), 491 (74%), and 405 (61%) completed the assessments when their children were 3, 4, and 5 years old. The intervention was significantly associated with a lower mean BMI: 15.90 (SE 0.08) vs. 16.20 (SE 0.08), difference −0.30 (95% CI: −0.59 to −0.01, *P* = 0.039). This association was stronger among low-income families, difference −0.57 (95% CI: −1.05 to −0.10, *P* = 0.018).

**Conclusions:**

The two-year telephone-based intervention was associated with decreased mean BMI of preschool-aged children. Telephone-based support for mothers could reduce obesity risk in preschool-aged children, particularly among low-income families.

**Trial registration:**

The original RCT is registered with the Australian Clinical Trial Registry (ACTRN12618001571268).

## Background

Preventing obesity in the first 2000 days of life is increasingly important given that there were 39 million children with overweight or obesity under the age of 5 years globally in 2020 [1]. Early prevention is critical because it not only helps prevent long-term health issues and improves the overall well-being of children but also shapes healthy lifestyle habits that will have a lifelong impact on the health of the whole population. Thus, the World Health Organization (WHO) Report of the Commission on Ending Childhood Obesity called for all stakeholders to take action to reduce obesity risk. Recommended actions [2] include guidance and support for a healthy diet, sleep, and physical activity in early childhood.

Evidence has suggested that early interventions targeting mothers in pregnancy and postpartum can improve young children’s healthy habits of feeding, eating, and actively playing, with mixed findings on body mass index (BMI) [3–5]. Hence, educating parents, creating supportive home and childcare environments, and developing healthy eating and physical activity policies are vital strategies for preventing early childhood obesity. Face-to-face education programs including home visiting [6], parenting classes or groups [7, 8], and childcare settings with direct parental involvement [9], can be effective in reducing obesity risk in young children. Several studies have explored the effectiveness of telephone-based interventions for obesity prevention in early life, with some evidence suggesting positive outcomes in improved feeding and eating habits and reduced obesity risk [10–12].

Telehealth is the use of telecommunication technologies such as educational text messages or phone calls, interactive voice response systems, smartphone applications, video chat, or online platforms to provide health care. It is increasingly used in public health interventions and health information dissemination [13, 14]. Telehealth gained popularity as an alternative for in-person healthcare and health promotion programs during the COVID-19 pandemic. However, evidence of the effectiveness of telephone and SMS based obesity interventions targeting preschool-aged children and carers is lacking. Only one 6-month intervention study using home visiting approach together with telephone calls and text messages showed significant association with decreased child BMI [15], while other studies did not find such an effect.

To fill this knowledge gap, in 2019–2020 we conducted a randomised controlled trial (RCT) using three-staged telephone and SMS intervention sessions to mothers with children aged 2 years. We found that the 12-month intervention improved dietary and activity behaviours at age 3 years but had no effect on child BMI except for children from lower income households [12]. The trial was to be concluded at the end of 2020 (child age 3 years) when Sydney was experiencing the first wave of COVID-19 cases. The second wave in mid-2021 involved large-scale lockdowns which resulted in the suspension of face-to-face healthcare services [16]. COVID created a need to extend the original trial to support participating families [17]. Therefore, by extending the original trial, we aimed to evaluate the effectiveness of a two-year telephone-based intervention on body mass index (BMI), eating habits, active play, and screen time behaviours among preschool-aged children.

## Methods

### Study design

The original RCT [12] tested the effectiveness of telephone-based early obesity intervention for children aged 2–3 years (in 2019–20) in Sydney, Australia. The original study protocol [18] and main findings can be found elsewhere [12]. For this extension study, we implemented two staged intervention sessions from 3 to 4 years old (i.e. at 38–40 and 42–44 months of child age) in 2020–21, with further one year follow-up (no intervention) from 4 to 5 years old in 2021–22. The original study protocol and trial registration were amended prior to commencement of this study.

### Setting, participants and recruitment

At the end of the original trial (child aged 3 years), we obtained consent from the participating mothers to continue participating in the study with a letter of invitation and information about this extension study. The detailed recruitment process of the original trial has been reported elsewhere [12].

### Eligibility criteria

Mother-child dyads were eligible for this extension study if they had participated in the original trial at child ages 2–3 years with no major health issues reported.

### Randomisation

The group allocation remained the same according to their previous randomisation allocation in the original trial. The detailed randomisation of the original trial was reported elsewhere [12].

### Intervention

In this extension study we implemented two telephone-based intervention sessions at 38–40 and 42–44 months of child age in addition to three intervention sessions delivered in the original trial. Briefly, the intervention model was informed by the Health Belief Model [19], and previous research [10–12]. Each staged intervention period started with a mailout of an intervention booklet (Supplementary Document 1), followed by a telephone support call and SMS. The telephone support calls, lasting 45–60 min, were made by Child and Family Health Nurses to support mothers regarding their child’s healthy eating, nutrition, and physical activity (Supplementary Document 2). Due to the COVID-19 pandemic, the telephone support sessions were longer than planned to give mothers the opportunity to discuss COVID-19 related issues such as measures of protection and immunisation against COVID-19. We used a two-way automated SMS system to send SMSs twice a week for four weeks at a predetermined time (10 am–1 pm). A full list of SMS text messages can be viewed via Supplementary Document 3.

### Control

In this extension study, as a retention strategy, we sent out two information packages with information not related to the intervention such as toilet training, language development and COVID-19 information.

### Main measures and outcomes

We collected data at ages 4 and 5 years using the same data collection methods and tools as used for the 3-year outcomes [12]. As per 3-year outcome measures, we also contracted the same independent survey company for data collection using a computer-assisted telephone interview.

The primary outcome was child BMI. For weight and height measurements, we mailed out anthropometry measurement kits [12], along with detailed instructions to participating families 3 weeks prior to their scheduled data collection at ages 4 and 5 years. We then asked mothers to report their child’s weight and height via telephone interviews. We used the WHO Anthro program software to obtain BMI values and BMI z-scores at ages 4 and 5 years.

Secondary outcomes were child screen time, outdoor play time and dietary behaviours (including consumption of fruit/vegetable, fast food, soft drink, and having a meal in front of the TV) reported by mothers through telephone interviews. The questionnaire and coding frame can be viewed in the supplementary statistical analysis plan. Families’ socio-demographic information was collected and reported at baseline of the original trial (Supplementary Table 1).

#### Intervention engagement

To monitor participant satisfaction and intervention engagement, a process evaluation was conducted, and findings will be reported elsewhere. The number of telephone support sessions held, or SMS messages sent was also recorded.

### Sample size

The sample size was estimated based on the original trial [12] and findings from a US study that detected a decrease in BMI of 0.40 kg/m2 in children aged 2–5 years [15]. A sample of 506 (253 per group) at ages 3–5 years would allow us to detect a difference of 0.40 kg/m^2^ in mean BMI (SD = 1.60) at the 2-sided 5% significance level with 80% power.

### Blinding

The data collectors employed by a market survey company for data collection using telephone interviews were blinded to the research hypotheses and intervention allocation.

#### Statistical analysis

For this study we adopted a statistical analysis plan developed for the original trial [12]. Briefly, we applied the intention-to-treat principle for all data analyses. We used multiple imputation (MI) with chained equations to impute missing values to address potential bias caused by missing data and loss to follow-up. We also conducted complete-case analyses as a form of sensitivity analysis. Using descriptive analysis, mean and standard error (SE) were reported for child BMI and BMI z-score and number and percentage were reported for binary outcomes.

We examined the patterns and mechanisms of missing data before generating MI datasets. We ran models for missingness examining whether missing was at random and used Little’s test to examine whether missing was completely at random. Since the missingness was not completely at random in this study, we used MI by chained equations to generate the MI datasets. We imputed all missing values for the full intention-to-treat analysis of all 662 randomized participants at baseline (2 years of age). To predict missing values, the imputation model included all plausible observed values of primary and secondary outcomes from 3 to 5 years of age and family demographics. We used 20 imputations to achieve a relative efficiency of 99% [20].

Since child BMI, BMI z-score, and secondary outcomes were measured repeatedly at 3 (i.e. the original trial), 4, and 5 years of age, to consider correlations between repeated measures, longitudinal analyses were conducted to ascertain the intervention effects from 3 to 5 years of age. Longitudinal models were conducted for outcomes from ages 3 to 4 years, and from ages 3 to 5 years to explore the immediate and longer-term effects. The interaction between time and intervention allocation was tested. For child BMI and BMI z-score, random intercept mixed linear models were fitted. For binary outcomes, such as dietary behaviour, random intercept mixed logistic models were fitted. Since an interaction between intervention and household income was detected in the original trial at 3 years [12], a subgroup analysis by household income was also conducted in this study for child BMI and BMI z-score. All regression models were adjusted for previous intervention exposure of the participants (in their late pregnancy to 2 years of age [11]). Adjusted coefficient (β) with 95% confidence interval (CI) and adjusted odds ratios (AORs) with 95% CI were reported for continuous (e.g. child BMI and BMI z-score) and binary outcomes respectively. All statistical analyses were performed using statistical software Stata version 16 (StataCorp, 2016). All *P*-values were two-sided, and statistical significance was set at the 5% level.

## Results

### Baseline characteristics and follow-up

**Fig. 1:**
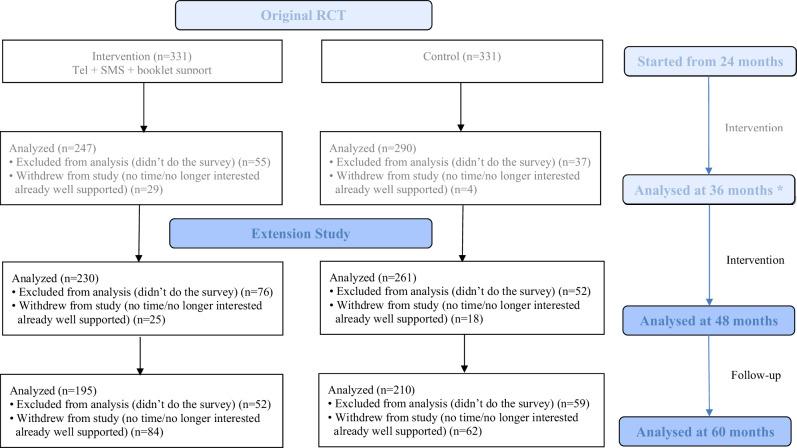
CONSORT diagram. Wen LM, Xu H, Phongsavan P, et al. Twelve-month effectiveness of telephone and SMS support to mothers with children aged 2 years in reducing children’s BMI: a randomized controlled trial. International Journal of Obesity. 2023; 10.1038/s41366-023-01311-7.

Figure 1 shows that 662 mother-child dyads recruited in the original trial were randomised into intervention (*n* = 331) or control (*n* = 331) at age 24 months. We previously reported baseline characteristics of the study participants in the 36-month outcome paper [12] (Supplementary Table 1). For this extension study, at child aged 4 and 5 years, 230 (69.5%) and 195 (58.9%) remained in the intervention while 261 (78.9%) and 210 (63.4%) remained in the control, respectively. As with the original trial, mothers were more likely to be lost to follow-up if they were younger, unemployed, or had lower income or education levels. However, characteristics of those participants who remained at 4 and 5 years were similar between the groups (Supplementary Table 2).

#### Primary outcome

Table 1 shows comparisons of means (SE, mean diff 95% CIs) of child BMI and BMI z-score between the groups on longitudinal analysis with multiple imputations. For ages 3–4 years, a mean BMI difference was observed but was not significant. For ages 3–5 years, the mean BMI was 15.90 kg/m^2^ (SE 0.08) for the intervention group and 16.20 kg/m^2^ (SE 0.08) for the control group, with a difference of −0.30 (95% CI −0.59 to −0.01). In subgroup analysis of families with annual household income <$80,000, for ages 3–5 years, the mean BMI was 15.76 kg/m^2^ (SE 0.14) for the intervention group and 16.34 kg/m^2^ (SE 0.12) for the control group, with a difference of −0.57 (95% CI −1.05 to −0.10). These differences in reduced mean BMI comparing the intervention with the control were evident for both 3–4 years and 3–5 years on complete-case analysis as shown in Supplementary Table 3.

**Table 1:** Comparisons of primary outcomes between intervention and control groups from 3 to 4 years, and 3 to 5 years of age using longitudinal analysis (intention-to-treat analysis with multiple imputations) and subgroup analysis.

Primary outcomes	Intervention mean (SE)	Control mean (SE)	Intervention – control mean difference (95% CI)^a^	*P*
Outcomes from 3 to 4 years of age				
Multiple imputation *n* = 662	*n* = 331	*n* = 331		
BMI	16.11 (0.08)	16.37 (0.09)	−0.26 (−0.54 to 0.02)	0.073
BMI z-score	0.43 (0.06)	0.61 (0.06)	−0.18 (−0.38 to 0.02)	0.079
Subgroup analysis *n* = 662				
Annual household income <$ 80,000 *n* = 256	*n* = 124	*n* = 132		
BMI	16.03 (0.15)	16.51 (0.15)	−0.48 (−0.97 to 0.00)	0.052
BMI z-score	0.33 (0.11)	0.68 (0.10)	−0.35 (−0.69 to −0.01)	0.044
Annual household income ≥$ 80,000 *n* = 406	*n* = 207	*n* = 199		
BMI	16.17 (0.10)	16.27 (0.10)	−0.10 (−0.45 to 0.24)	0.551
BMI z-score	0.48 (0.07)	0.55 (0.07)	−0.07 (−0.31 to 0.18)	0.595
Outcomes from 3 to 5 years of age				
Multiple imputation *n* = 662	*n* = 331	*n* = 331		
BMI	15.90 (0.08)	16.20 (0.08)	−0.30 (−0.59 to −0.01)	0.039
BMI z-score	0.29 (0.06)	0.49 (0.05)	−0.20 (−0.40 to −0.01)	0.045
Subgroup analysis *n* = 662				
Annual household income <$ 80,000 *n* = 256	*n* = 124	*n* = 132		
BMI	15.76 (0.14)	16.34 (0.12)	−0.57 (−1.05 to −0.10)	0.018
BMI z-score	0.17 (0.09)	0.57 (0.09)	−0.40 (−0.72 to −0.07)	0.018
Annual household income ≥$ 80,000 *n* = 406	*n* = 207	*n* = 199		
BMI	15.99 (0.10)	16.11 (0.10)	−0.12 (−0.48 to 0.24)	0.500
BMI z-score	0.37 (0.07)	0.45 (0.07)	−0.08 (−0.33 to 0.17)	0.523

^a^Mean differences from multiple mixed linear regression models adjusted previous intervention allocation.

#### Secondary outcomes

On longitudinal analysis with multiple imputations, Table 2 compares secondary outcomes in percentage or AORs between intervention and control for children aged 3–4 years. The intervention was significantly associated with not eating in front of TV (79% in intervention vs. 66% in control, AOR 3.22, 95% CI 1.72–6.01), as well as meeting four or more recommendations for dietary behaviours (63% in intervention vs. 55% in control, AOR 1.80, 95% CI 1.10–2.96). Similarly, Table 3 also shows the outcome comparisons between the groups for children aged 3–5 years. Outcome improvements were observed in not having fast food (29% in intervention vs. 23% in control, AOR 1.63, 95% CI 1.01–2.65), and not eating in front of TV (77% in intervention vs. 65% in control, AOR 2.65, 95% CI 1.57–4.67) as well as meeting four or more recommendations for dietary behaviours (57% in intervention vs. 50% in control, AOR 1.70, 95% CI 1.09–2.65). These findings were similar to those found in complete case analysis (Supplementary Tables 4, 5).

**Table 2:** Comparisons of secondary outcomes of child and mother between intervention and control groups from 3 to 4 years of age: longitudinal analysis (intention-to-treat analysis with multiple imputations).

Secondary outcomes	Intervention total = 331 *n* (%)	Control total = 331 *n* (%)	Intervention vs. control AOR (95% CI)
Fruit consumption			
Meet fruit recommendation	280 (85)	289 (87)	0.73 (0.43–1.25)
Vegetable consumption			
Meet vegetable recommendation	65 (20)	52 (16)	1.84 (0.88–3.82)
Fast food			
No	112 (34)	93 (28)	1.57 (0.95–2.59)
Soft drink			
No	265 (80)	266 (80)	0.98 (0.52–1.83)
Food for reward			
No	271 (82)	252 (76)	1.59 (0.96–2.63)
Eat in front of TV			
No	261 (79)	217 (66)	3.22 (1.72–6.01) *P* < 0.0001
Dietary behaviour			
Meet all 4 or more recommendations	207 (63)	181 (55)	1.80 (1.10–2.96) *P* = 0.020
Outdoor playtime			
≥2 h/day	219 (66)	218 (66)	1.01 (0.65–1.58)
Screen time			
Meet screen time recommendation	120 (36)	106 (32)	1.30 (0.87–1.94)
Daily sleep duration			
≥10 h/day	284 (86)	276 (83)	1.30 (0.83–2.03)
Movement behaviour			
Meet all 3 recommendations	73 (22)	68 (20)	1.14 (0.73–1.77)

Meet fruit consumption recommendation: ≥1 serves/day at 3 years, ≥1.5 serves/day at 4 years.

Meet vegetable consumption recommendation: ≥2.5 serves/day at 3 years, ≥4.5 serves/day at 4 years.

Meet screen time guideline: ≤1 h/day at 3 and 4 years.

*AOR* adjusted odds ratio, adjusted for previous intervention allocation.

**Table 3:** Comparisons of secondary outcomes of child and mother between intervention and control groups from 3 to 5 years of age: longitudinal analysis (intention-to-treat analysis with multiple imputations).

Secondary outcomes	Intervention total = 331 *n* (%)	Control total = 331 *n* (%)	Intervention vs. Control AOR (95% CI)
Fruit consumption			
Meet fruit recommendation	273 (83)	284 (86)	0.71 (0.47–1.08)
Vegetable consumption			
Meet vegetable recommendation	46 (14)	38 (12)	1.46 (0.89–2.41)
Fast food			
No	95 (29)	76 (23)	1.63 (1.01–2.65) *P* = 0.047
Soft drink			
No	258 (78)	255 (77)	1.12 (0.60–2.08)
Food for reward			
No	276 (82)	259 (78)	1.36 (0.87–2.12)
Eat in front of TV			
No	253 (77)	215 (65)	2.65 (1.57–4.67) *P* < 0.0001
Dietary behaviour			
Meet all 4 or more recommendations	190 (57)	165 (50)	1.70 (1.09–2.65) *P* = 0.020
Outdoor playtime			
≥2 h/day	221 (67)	218 (66)	1.06 (0.75–1.49)
Screen time			
Meet screen time recommendation	144 (44)	127 (38)	1.34 (0.97–1.85)
Daily sleep duration			
≥10 h/day	279 (84)	267 (80)	1.40 (0.94–2.09)
Movement behaviour			
Meet all 3 recommendations	85 (26)	75 (23)	1.25 (0.90–1.74)

Meet fruit consumption recommendation: ≥1 serves/day at 3 years, ≥1.5 serves/day at 4 and 5 years.

Meet vegetable consumption recommendation: ≥2.5 serves/day at 3 years, ≥4.5 serves/day at 4 and 5 years.

Meet screen time guideline: ≤1 h/day at 3 and 4 years, ≤2 h/day at 5 years.

*AOR* adjusted odds ratio, adjusted for previous intervention allocation.

### Intervention engagement

Of 331 mothers in the intervention group, 213 mothers (64%) received an intervention telephone session at 38–40 months of child age, while just 192 (58%) received the session at 42–44 months of child age. In addition to telephone support, automatic SMS messages were sent, but the scheduling SMS system does not allow us to identify whether mothers read the messages or not.

## Discussion

### Principal findings of the study

This extension study found that the five-staged intervention consisting of telephone calls and SMS-support plus mailed intervention booklets between children aged 2–4 years was significantly associated with reduced mean BMI of children aged up to 5 years. The association was more evident among children from low-income families. The intervention was also significantly associated with improved eating habits and meeting dietary recommendations of participating children. But the intervention was not significantly associated with improvements in child active play and screen time behaviours.

### Comparing the study findings with previous research

A 2016 systematic review by Ling et al. [5] on obesity prevention and management of preschool children demonstrated that more than 60% of preventive interventions resulted in little to no improvement of BMI or BMI z-scores. However, eight preventive interventions had significant effects on anthropometric measures. Half of these were theory-based, three of which were grounded in social cognitive theory. The effective interventions were mainly school-based, utilised parental involvement, targeted both physical activity and nutrition, and had a mean intervention duration of 9.63 months. Similarly, Brown et al.’s 2019 Cochrane systematic review [3] identified that targeting a combination of nutrition and physical activity was the most effective in lowering BMI in 0–5-year-olds. Furthermore, longer interventions have shown promising results in lowering anthropometric measures, such as the effect of Sharma et al.’s two-year study [21] on reducing child BMI scores. Shorter interventions may overlook delayed intervention effects [5]. However, the beneficial effects of interventions can also dissipate over time after an intervention ends [22, 23]. Therefore, limits to the duration and dose of interventions can contribute to their lack of efficacy. Our intervention was spread over a 24-month period (child ages 2–4 years) with three years of follow-up outcome assessments. All these factors may have contributed to the statistically significant effects of our intervention on child BMI and BMI z-scores. In contrast, the 12-month intervention (i.e., 2–3 years) of the original trial found no significant overall effect on BMI except among children from lower household income families.

Our findings on dietary and movement behaviours were generally in line with the findings from a previous systematic review of mobile health interventions targeting parents to prevent and treat childhood obesity [14]. In this review, nineteen studies (14 RCTs and 5 non-randomised trials) were included but only one study reported a significant increase in physical activity and six interventions proved to be effective in changing dietary behaviours, while other studies observed no effect. The possible reasons why certain behaviours changed and not others as a result of a particular intervention could be complex and require further research.

### Telehealth intervention

Telehealth has been increasingly utilised as a modality to treat patients virtually, particularly with the development of the COVID-19 pandemic and implementations of social distancing. A 2021 systematic review [24] identified telehealth to be the most clinically effective in the disciplines of psychiatry and endocrinology and demonstrated that telehealth can be as clinically effective as face-to-face care.

Our study similarly identified telehealth as effective in preventing child obesity by lowering mean BMI of preschool-aged children. Therefore, telehealth has played an important role in not only clinical settings, but also has potential of sustaining health promotion and research. While we found telehealth to be effective, other telephone-based clinical trials investigating the effect of telehealth on paediatric obesity did not demonstrate a significant effect in decreasing child BMI scores overall [13, 14, 25, 26]. Two systematic reviews [13, 14] on telehealth interventions delivered to children revealed an overall lack of robust effect on improvement of anthropometric measures. However, similar to our study, positive effects on dietary behaviours were identified in several interventions. Notably, Barefield et al.’s review [13] found that interventions supplemented with motivational coaching resulted in a marked reduction in BMI or BMI z-scores, suggesting that telehealth modalities alone may not be efficacious enough. Our study provided motivational interviewing, which may be one of the contributing factors to the significant effects we found on BMI. Hence, telehealth interventions may require supplementary services, such as in-person sessions or motivational coaching, to enhance their effectiveness. Nevertheless, the potential for telehealth to address child obesity prevention is promising.

### Subgroup analysis

This study identified a post-intervention reduction in BMI and BMI z-scores of preschool-aged children from low-income families through subgroup analysis. Currently, there is limited evidence that obesity interventions are more effective in lower-income families. A systematic review and meta-analysis [27] of obesity treatment for children aged 6–18 found a similar significant subgroup difference, where interventions in lower-middle income groups were found to have greater effects on BMI and BMI z-scores than high-income groups. Moreover, studies such as the Healthy Habits, Healthy Homes intervention [15], which targeted a low-income population, also found a significant decrease in BMI in post-intervention children. There are several reasons why such interventions may be more effective in a low-income, minority population. Studies have shown that disadvantaged, low-income, or minority background families have an increased prevalence of obesity compared to their counterparts [28–30]. We assume that the disadvantaged and low-income families are usually less likely to get input from health services or credible health messages due to potential barriers for accessing health services such as language barrier, thus getting clear evidence-based advice makes a greater difference. They may also have had less opportunity to participate in health promotion programs that are tailored for them, so the impact may be greater as observed in this study.

### Major gaps identified in this area and implications of practice

Currently, there is still limited evidence on the effects of telehealth interventions on obesity outcomes of children in the first 2000 days. Existing studies have limited generalisability to other populations, particularly due to varying telehealth modalities and study populations. There is an increasing need for future interventions to be tailored towards risk populations, such as low-income, minority, or culturally and linguistically diverse backgrounds. Moreover, these differences in study design and population limit the ability to make meaningful comparisons between studies, and the ability to evaluate intervention characteristics that are particularly effective in preventing childhood obesity. Research on preventive telehealth interventions is also limited, with most studies involving management of children already with overweight or obesity [5]. Our study demonstrates the potential for telehealth in a preventive framework to improve obesity risk outcomes of preschool-aged children, particularly low-income children, and future research into this space is warranted.

This research has several implications. The strong effect of the telehealth intervention on child BMI among low-income families should be further investigated. The specific telehealth modalities and population characteristics that telehealth is effective in addressing should be elucidated in further research. Our research demonstrates the potential for telehealth services to be utilised in future clinical practice surrounding obesity prevention and health promotion. Future development of telehealth programs targeting disadvantaged low-income communities has the capacity to address both national and global concerns surrounding childhood obesity. Further work is needed in conducting and investigating the cost-effectiveness of such programs. The inclusion of telehealth modalities within current child obesity policies has great potential in shaping future healthcare promotion and practices within a world post COVID-19. The results demonstrate that telephone support can be an alternative approach to widely used face-to-face approaches in promoting child health, particularly if face-to-face health service contacts are restricted temporarily. Our results also underscore the importance of longer periods of intervention and follow-up of the outcome measures.

#### Strengths and limitations

The strengths of this study include the design of a randomised controlled trial [12]. The intervention was guided by the health promotion theory and well informed by previous studies [10–12]. The primary outcome of BMI (weight and height) was objectively measured by mothers using a standardised toolkit, and secondary outcomes were assessed using validated questionnaires by blinded interviewers of the market survey company. We conducted longitudinal data analysis with multiple measures of the same child over a three-year time span (ages 3–5 years). We also applied the intention to treat principal with MI and used a multilevel approach to consider correlations between repeated measures on the same children in data analysis.

However, this study had some limitations. First, the generalisability of the study findings could be limited due to the study location and participant demographics. Second, although we used MI to address potential bias caused by missing data and loss to follow-up, a relatively high loss to follow-up rate was noticeable. Third, due to disruptions caused by the COVID-19 pandemic, the trial intervention and data collection were conducted pragmatically rather than ideally. The potential impact of COVID-19 on the trial delivery and outcomes could not be ignored. In addition, secondary outcomes were limited by mothers’ self-reporting.

## Conclusion

By extending the original RCT to the age of 5 years, this study found that an early staged intervention using nurse-led telephone and SMS support was significantly associated with reduced BMI in preschool-aged children, particularly those from lower income families. The intervention was also significantly associated with improved dietary behaviours but was not associated with children’s play activity and screen time behaviours. Telephone-based support together with SMS for mothers has a great potential in reducing obesity risk in preschool-aged children.

## Supplementary information

Supplementary Tables

Supplementary Document 1_3-4 years Booklet 1

Supplementary Document 1_3-4 years Booklet 2

Supplementary Document 2_3-4 years_Tel 1

Supplementary Document 2_3-4 years_Tel 2

Supplementary Document 3_3-4 years_SMS

## Data Availability

De-identified data and material can be available on request pending on ethics approval.
