# Supplementary material for: Effectiveness of a telephone-based randomised clinical trial targeting obesity risk of preschool-aged children: An extension study during the COVID-19 pandemic
Source: Int J Obes (Lond). 2025 Aug 14;49(11):2241–8. doi: 10.1038/s41366-025-01869-4 (PMC12583190; doi:10.1038/s41366-025-01869-4)
Supplement: Supplementary file 1 — Supplementary Tables [file 41366_2025_1869_MOESM1_ESM.docx]

**Supplementary Tables**

sTable 1: Mothers’ characteristics, child BMI and BMI z-score at baseline by group allocation

| **Variables** | **Total**  **N=662**  **n (%)** | **Intervention**  **n=331**  **n (%)** | **Control**  **n=331**  **n (%)** | **P^*^** |
| --- | --- | --- | --- | --- |
| **Mother’s age** |  |  |  | 0.099 |
| < 30 years | 183 (28) | 101 (31) | 82 (25) |  |
| ≥ 30 years | 479 (72) | 230 (69) | 249 (75) |  |
| **Country of birth** |  |  |  | 0.810 |
| Australia | 251 (38) | 124 (37) | 127 (38) |  |
| Other | 411 (62) | 207 (63) | 204 (62) |  |
| **Language spoken at home** |  |  |  | 0.586 |
| English | 351 (53) | 179 (54) | 172 (52) |  |
| Other | 311 (47) | 152 (46) | 159 (48) |  |
| **Annual household income** |  |  |  | 0.495 |
| < $ 80.000 | 257 (39) | 124 (37) | 133 (40) |  |
| ≥ $ 80,000 | 405 (61) | 207 (63) | 198 (60) |  |
| **Employment status** |  |  |  | 0.869 |
| Employed | 444 (67) | 223 (67) | 221 (67) |  |
| Other | 218 (33) | 108 (33) | 110 (33) |  |
| **Marital status** |  |  |  | 0.372 |
| Married/de-facto partner | 629 (95) | 317 (96) | 312 (94) |  |
| Other | 33 (5) | 14 (4) | 19 (6) |  |
| **Education level** |  |  |  | 0.205 |
| Up to HSC* to TAFE^/Diploma | 201 (30) | 108 (33) | 93 (28) |  |
| University | 461 (70) | 223 (67) | 238 (72) |  |
| **Father’s employment status** |  |  |  | 0.550 |
| Employed | 601 (91) | 303 (92) | 298 (90) |  |
| Other | 61 (9) | 28 (8) | 33 (10) |  |
| **Father’s education level** |  |  |  | 0.218 |
| Up to HSC* to TAFE^/Diploma | 253 (38) | 119 (36) | 134 (41) |  |
| University | 409 (62) | 212 (64) | 197 (59) |  |
| **First time mother** |  |  |  | 0.389 |
| No | 293 (44) | 152 (46) | 141 (43) |  |
| Yes | 369 (56) | 179 (54) | 190 (57) |  |
|  | **Mean (SD)** | **Mean (SD)** | **Mean (SD)** | **P^#^** |
| **Child BMI** | 16.91 (1.52) | 16.83 (1.44) | 17.00 (1.59) | 0.131 |
| **Child BMI z-score** | 0.85 (1.03) | 0.79 (0.98) | 0.91 (1.07) | 0.139 |

*HSC: Higher School Certificate (Year 12), ^TAFE: Technical and Further Education

P^*^: P value of Pearson’s Chi-squared test

P^#^: P value of Student’s t-test

sTable 2: Mothers’ baseline characteristics by group allocation at 4- and 5-years follow-up (complete case analysis)

| **Variables** | **4 years** | | | **5 years** | | |
| --- | --- | --- | --- | --- | --- | --- |
|  | **Intervention**  **n=230**  **n (%)** | **Control**  **n=261**  **n (%)** | **P** | **Intervention**  **n=195**  **n (%)** | **Control**  **n=210**  **n (%)** | **P** |
| **Mother’s age** |  |  | 0.152 |  |  | 0.263 |
| < 30 years | 64 (28) | 58 (22) |  | 54 (28) | 48 (23) |  |
| ≥ 30 years | 166 (72) | 203 (78) |  | 141 (72) | 162 (77) |  |
| **Country of birth** |  |  | 0.829 |  |  | 0.885 |
| Australia | 85 (37) | 94 (36) |  | 72 (37) | 79 (38) |  |
| Other | 145 (63) | 167 (64) |  | 123 (63) | 131 (62) |  |
| **Language spoken at home** |  |  | 0.490 |  |  | 0.988 |
| English | 127 (55) | 136 (52) |  | 106 (54) | 114 (54) |  |
| Other | 103 (45) | 125 (48) |  | 89 (46) | 96 (46) |  |
| **Annual household income** |  |  | 0.420 |  |  | 0.173 |
| < $ 80.000 | 142 (66) | 148 (63) |  | 59 (33) | 76 (40) |  |
| ≥ $ 80,000 | 72 (34) | 88 (37) |  | 121 (67) | 116 (60) |  |
| **Employment status** |  |  | 0.875 |  |  | 0.748 |
| Employed | 154 (67) | 173 (66) |  | 132 (68) | 139 (66) |  |
| Other | 76 (33) | 88 (34) |  | 63 (32) | 71 (34) |  |
| **Marital status** |  |  | 0.261 |  |  | 0.764 |
| Married/de-facto partner | 221 (96) | 244 (94) |  | 186 (95) | 198 (95) |  |
| Other | 9 (4) | 16 (6) |  | 9 (5) | 11 (5) |  |
| **Education level** |  |  | 0.286 |  |  | 0.443 |
| Up to HSC* to TAFE^/Diploma | 158 (69) | 190 (73) |  | 137 (70) | 154 (74) |  |
| University | 72 (31) | 70 (27) |  | 58 (30) | 55 (26) |  |
| **Father’s employment status** |  |  | 0.556 |  |  | 0.987 |
| Employed | 209 (92) | 230 (91) |  | 178 (92) | 188 (92) |  |
| Other | 18 (8) | 24 (9) |  | 16 (8) | 17 (8) |  |
| **Father’s education level** |  |  | 0.253 |  |  | 0.494 |
| Up to HSC* to TAFE^/Diploma | 145 (65) | 153 (60) |  | 124 (65) | 127 (62) |  |
| University | 77 (35) | 101 (40) |  | 66 (35) | 78 (38) |  |
| **First time mother** |  |  | 0.887 |  |  | 0.215 |
| No | 99 (43) | 114 (44) |  | 80 (41) | 99 (47) |  |
| Yes | 131 (57) | 147 (56) |  | 115 (59) | 111 (53) |  |

*HSC: Higher School Certificate (Year 12), ^TAFE: Technical and Further Education, P*: P value of Pearson’s Chi-squared test

sTable 3: Comparisons of primary outcomes between intervention and control groups (complete case analysis)

| **Primary outcomes** | **Intervention**  **Mean (SE)** | **Control**  **Mean (SE)** | **Intervention - Control**  **Mean difference (95% CI)^*^** | **P** |
| --- | --- | --- | --- | --- |
| **Outcomes from 3 to 4 years of age** |  |  |  |  |
| **BMI** | 16.14 (0.26) | 16.39 (0.28) | -0.25 (-0.49 to -0.01) | 0.041 |
| **BMI z-score** | 0.46 (0.14) | 0.63(0.14) | -0.17 (-0.34 to -0.01) | 0.048 |
| **Subgroup analysis** |  |  |  |  |
| **Annual household income <$ 80,000** |  |  |  |  |
| **BMI** | 16.10 (0.12) | 16.68 (0.25) | -0.58 (-1.05 to -0.11) | 0.016 |
| **BMI z-score** | 0.40 (0.04) | 0.82 (0.12) | -0.42 (-0.75 to -0.09) | 0.012 |
| **Annual household income ≥$ 80,000** |  |  |  |  |
| **BMI** | 16.25 (0.28) | 16.27 (0.27) | -0.02 (-0.30 to 0.26) | 0.887 |
| **BMI z-score** | 0.55 (0.15) | 0.56 (0.14) | -0.01 (-0.19 to 0.20) | 0.974 |
| **Outcomes from 3 to 5 years of age** |  |  |  |  |
| **BMI** | 15.92 (0.27) | 16.25 (0.24) | -0.30 (-0.51 to -0.09) | 0.005 |
| **BMI z-score** | 0.32 (0.16) | 0.53 (0.14) | -0.20 (-0.35 to -0.05) | 0.008 |
| **Subgroup analysis** |  |  |  |  |
| **Annual household income <$ 80,000** |  |  |  |  |
| **BMI** | 15.75 (0.37) | 16.54 (0.22) | -0.79 (-1.19 to -0.40) | <0.0001 |
| **BMI z-score** | 0.17 (0.23) | 0.71 (0.13) | -0.54 (-0.81 to -0.26) | <0.0001 |
| **Annual household income ≥$ 80,000** |  |  |  |  |
| **BMI** | 16.09 (0.24) | 16.11 (0.24) | -0.02 (-0.27 to 0.23) | 0.853 |
| **BMI z-score** | 0.45 (0.14) | 0.46 (0.14) | -0.01 (-0.18 to 0.18) | 0.969 |

^*^: Mean differences from multiple mixed linear regression models adjusted previous intervention allocation.

sTable 4: Comparisons of secondary outcomes of child and mother between intervention and control groups from 3 to 4 years of age (complete case analysis)

| **Secondary outcomes** | **Intervention^*^**  **n (%)** | **Control^#^**  **n (%)** | **Intervention vs. Control**  **AOR (95% CI)** |
| --- | --- | --- | --- |
| **Fruit consumption** |  |  |  |
| Meet fruit recommendation | 206 (87) | 244 (89) | 0.84 (0.57 – 1.25) |
| **Vegetable consumption** |  |  |  |
| Meet vegetable recommendation | 49 (20) | 46 (17) | 1.35 (0.96 – 1.91) |
| **Fast food** |  |  |  |
| No | 79 (33) | 79 (28) | 1.25 (0.96 – 1.63) |
| **Soft drink** |  |  |  |
| No | 198 (83) | 226 (82) | 1.10 (0.79 – 1.52) |
| **Food for reward** |  |  |  |
| No | 200 (84) | 214 (78) | 1.47 (1.07 – 2.02) |
| **Eat in front of TV** |  |  |  |
| No | 196 (82) | 185 (67) | 2.22 (1.66 – 2.98) |
| ***Dietary behaviour*** |  |  |  |
| *Meet all 4 or more recommendations* | 160 (67) | 159 (58) | 1.52 (1.17 – 1.97) |
| **Outdoor playtime** |  |  |  |
| ≥2 hours/day | 162 (68) | 183 (66) | 1.07 (0.82 – 1.39) |
| **Screen time** |  |  |  |
| Meet screen time recommendation | 93 (39) | 92 (34) | 1.27 (0.98 – 1.64) |
| **Daily sleep duration** |  |  |  |
| ≥10 hours/day | 207 (87) | 233 (85) | 1.23 (0.86 – 1.76) |
| ***Movement behaviour*** |  |  |  |
| *Meet all 3 recommendations* | 58 (24) | 61 (22) | 1.14 (0.85 – 1.52) |

^*^: Sample size for intervention group was 247 at 3 years and 230 at 4 years, respectively.

^#^: Sample size for control group was 290 at 3 years and 261 at 4 years, respectively.

AOR: adjusted odds ratio, adjusted for previous intervention allocation.

Meet fruit consumption recommendation: ≥1 serves/day at 3 years, ≥1.5 serves/day at 4 years.

Meet vegetable consumption recommendation: ≥2.5 serves/day at 3 years, ≥4.5 serves/day at 4 years.

Meet screen time guideline: ≤1 hour/day at 3 and 4 years.

sTable 5: Comparisons of secondary outcomes of child and mother between intervention and control groups from 3 to 5 years of age (Complete case analysis)

| **Secondary outcomes** | **Intervention^*^**  **n (%)** | **Control^#^**  **n (%)** | **Intervention vs. Control**  **AOR (95% CI)** |
| --- | --- | --- | --- |
| **Fruit consumption** |  |  |  |
| Meet fruit recommendation | 190 (85) | 222 (88) | 0.82 (0.63 – 1.12) |
| **Vegetable consumption** |  |  |  |
| Meet vegetable recommendation | 34 (15) | 33 (13) | 1.29 (0.93 – 1.78) |
| **Fast food** |  |  |  |
| No | 62 (28) | 60 (24) | 1.28 (1.01 – 1.63) |
| **Soft drink** |  |  |  |
| No | 182 (81) | 202 (80) | 1.14 (0.88 – 1.49) |
| **Food for reward** |  |  |  |
| No | 191 (85) | 204 (80) | 1.40 (1.06 – 1.85) |
| **Eat in front of TV** |  |  |  |
| No | 180 (80) | 169 (67) | 2.04 (1.60 – 2.60) |
| ***Dietary behaviour*** |  |  |  |
| *Meet all 4 or more recommendations* | 140 (63) | 136 (54) | 1.49 (1.20 – 1.85) |
| **Outdoor playtime** |  |  |  |
| ≥2 hours/day | 152 (68) | 136 (67) | 1.06 (0.85 – 1.32) |
| **Screen time** |  |  |  |
| Meet screen time recommendation | 105 (47) | 101 (40) | 1.34 (1.08 – 1.66) |
| **Daily sleep duration** |  |  |  |
| ≥10 hours/day | 193 (86) | 210 (83) | 1.30 (0.97 – 1.73) |
| ***Movement behaviour*** |  | | |
| *Meet all 3 recommendations* | 64 (29) | 63 (25) | 1.22 (0.97 – 1.55) |

^*^: Sample size for intervention group was 247 at 3 years, 230 at 4 years, and 195 at 5 years, respectively.

^#^: Sample size for control group was 290 at 3 years, 261 at 4 years, and 210 at 5 years, respectively.

AOR: adjusted odds ratio, adjusted for time and previous intervention allocation.

Meet fruit consumption recommendation: ≥1 serves/day at 3 years, ≥1.5 serves/day at 4 and 5 years.

Meet vegetable consumption recommendation: ≥2.5 serves/day at 3 years, ≥4.5 serves/day at 4 and 5 years.

Meet screen time guideline: ≤1 hour/day at 3 and 4 years, ≤2 hours/day at 5 years.
