# Supplementary material for: Effectiveness of a telephone-based randomised clinical trial targeting obesity risk of preschool-aged children: An extension study during the COVID-19 pandemic
Source: Int J Obes (Lond). 2025 Aug 14;49(11):2241–8. doi: 10.1038/s41366-025-01869-4 (PMC12583190; doi:10.1038/s41366-025-01869-4)
Supplement: Supplementary file 2 — Supplementary Document 1_3-4 years Booklet 1 [file 41366_2025_1869_MOESM2_ESM.pdf]

THE PRESCHOOL YEARS
3-5 YEARS

Booklet 1

Supporting your family during your child's preschool years

HEALTHY BEGINNINGS

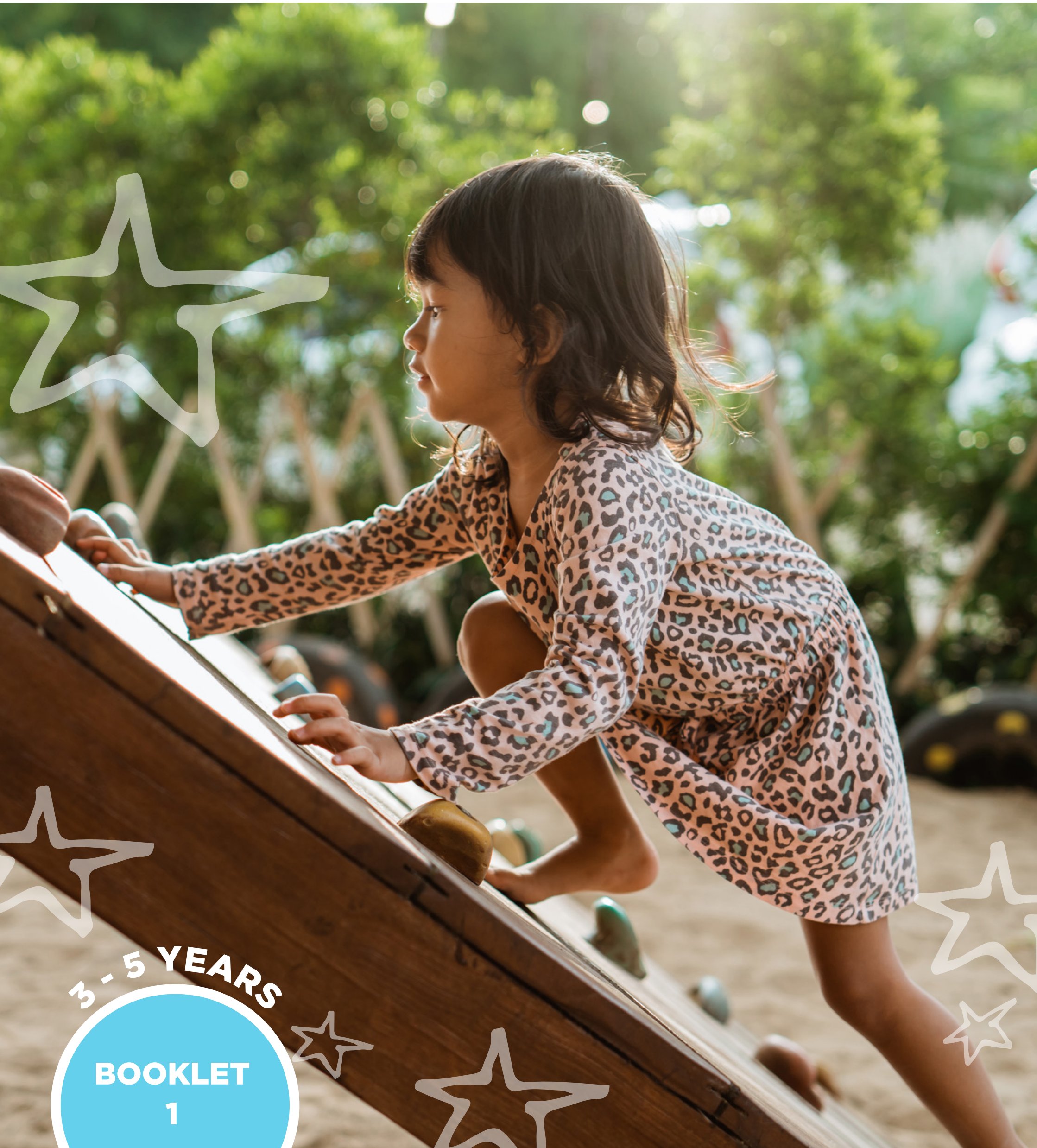

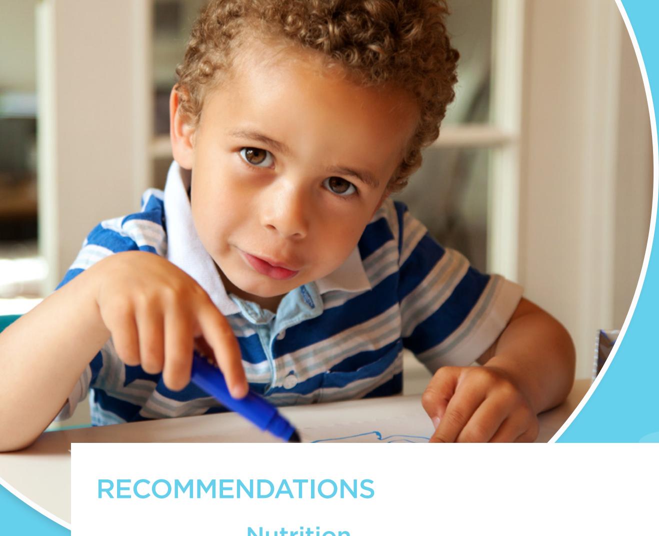

YOUR CHILD AT 3 - 5 YEARS

Healthy eating and active play are important for your preschooler (3 - 5 years).

This booklet provides information on your preschooler's health and tips for parents' health too.

RECOMMENDATIONS

Nutrition

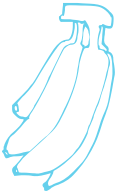

- It is best for your child to drink from a free-flow cup or an open cup and to stop all bottle feeds
- Drinks can include:
  - water
  - reduced fat cow's milk
  - soy or calcium enriched rice and oat milk can be used, but it is important to plan this with a health professional
- Preschoolers need lots of different healthy foods every day. This includes aiming for 2.5 serves of vegetables and 1 serve of fruit
- Keep breastfeeding, if you and your child are happy to keep going. For support, contact Australian Breastfeeding Association **www.breastfeeding.asn.au**

Active play

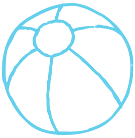

- **Lots of exercise and play:** Make sure your child spends at least 3 hours doing lots of different activities, every day
- **Staying active:** It's good for children to be active! Try to make sure they aren't still for longer than one hour at a time, unless they're sleeping
- **Screen time:** Between 3 - 5 years, children should be limited to less than 1 hour of screen time in a day with an adult watching or playing with them on an educational program or an app. Spending less time on the screen is better
- **Sleep:** 11 - 14 hours of good quality sleep every day. This can include night sleeps and naps

Immunisation

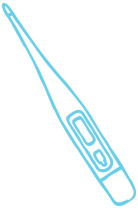

- Your child is due a 3 year Personal Health Record (blue book) developmental check so book an appointment with your Child and Family Health Nurse (CFHN) or GP to have this
- Your child's next immunisation is at 4 years Check **www.nsw.health.gov/immunisations** for more details
- Other immunisations are available - please discuss with your GP
- Your child is also due a yearly dental check – either with your family dentist of your local Community Health Dental Clinic

WHAT TO EXPECT AS A PARENT

From 3 – 5 years preschoolers will continue to go through developmental changes physically and emotionally. They will begin using words creatively to tell stories and will want to explore their independence as they are more active and can express their feelings. At this age most children are feeding themselves, using the toilet (although many have the occasional accidents) and they are able to wash themselves in the bath (under supervision).

YOUR PRESCHOOLER'S NUTRITION

What and how much food preschoolers need

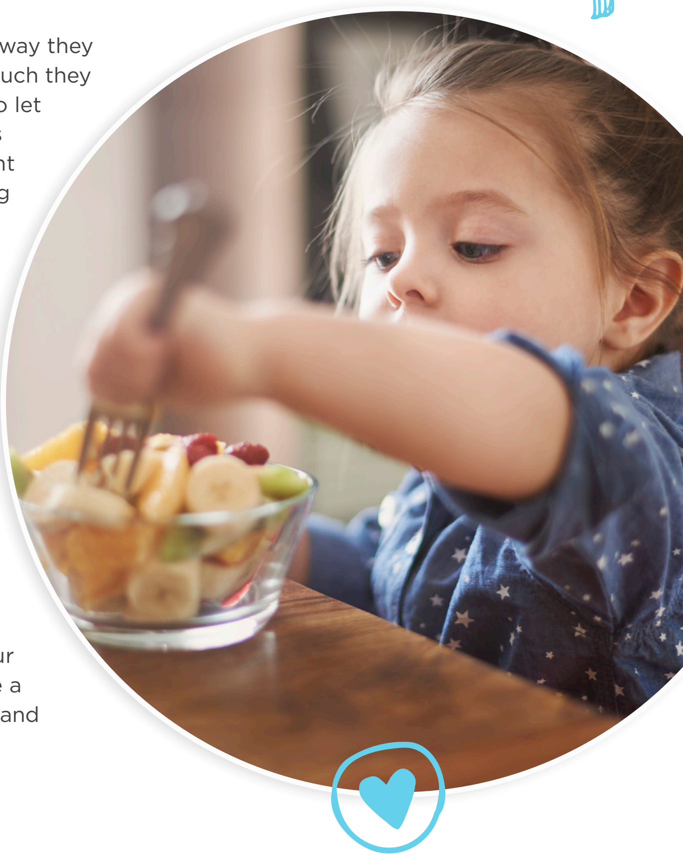

Every child is different with the way they eat, what they enjoy and how much they need. Following their appetite to let them decide how much to eat is important. At this age they might be able to express this by talking to you.

Preschoolers need the same variety of nutritious foods as older kids and adults - just in smaller amounts. When you understand what your child needs to grow well and be healthy, feeding them can become less frustrating.

The serving size (the amount of food) children at 3 – 5 years should have is about half an adult's serve. So the portion size on their plate at each meal will be about half the size of your plate. The recommendations are a general guideline based on age and activity level.

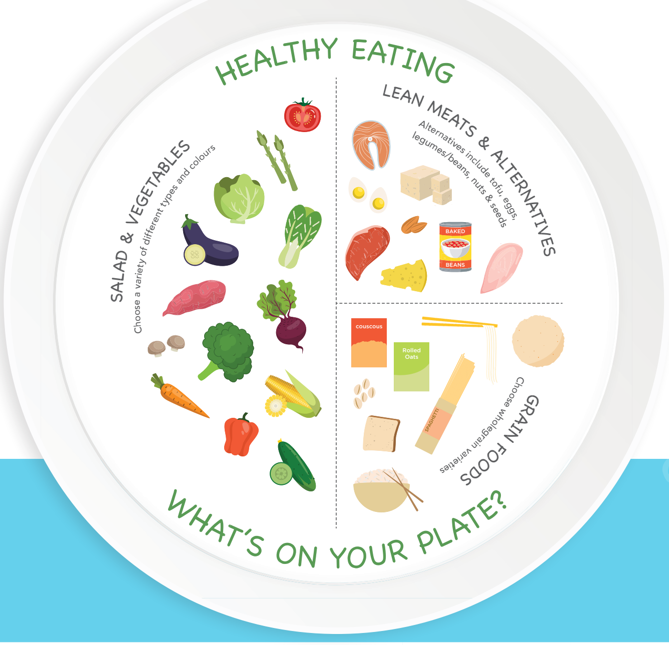

Food Group	Daily serving size	
	Children (3 - 5 years)	Adults
Vegetables and legumes/beans	2 1/2	5 1/2
Fruit	1	2
Grains	4	7
Lean meats and poultry, fish, eggs, tofu, nuts and seeds, and legumes/beans	1	2 1/2
Milk, yoghurt, cheese and/or alternatives, mostly reduced fat	1 1/2	3 1/2

Tips on how to feed your child a variety of food each day:

- Grains: 1 or 2 slices of wholegrain bread plus 1 cup cooked rice or pasta
- Vegetables: 1 – 1 ½ cups of raw or cooked vegetables including dark green, red and orange ones: mashed sweet potatoes, boiled broccoli or carrots with yoghurt dip or tomato sauce for pasta
- Fruit: 1 – 1 ½ cups of fresh or frozen fruits. Limit giving your child fruit juice as having whole fruits is better for their health. Kids love melon balls, sliced banana or strawberries and mandarin or oranges
- Dairy: 1 cup of reduced fat cow's milk or ¾ cup low fat yoghurt plus 1 slice of cheese
- Protein: 2 eggs or 1 small can of tuna or 65g lean red meat or 80g chicken or 100g fish

How to know if your child is eating the right amount?

Children develop different eating habits throughout their life which they learn from their environment. As long as they are growing well, be confident that they will eat to their hunger which is what they need to sleep, learn and be active. If you are worried that your child is eating too much or not enough, contact your GP, CFHN or a dietitian who can support you.

Tips to help your preschooler develop healthy eating habits:

- Eat meals together as a family as often as possible, or at least once a day
- If your child doesn't eat part of the meal – for example, the vegetables, this is their choice. You don't need to offer extra serves of other food to make up for not eating their vegetables
- Avoid distractions like TV or toys during mealtimes. This will help your child focus on their appetite
- Try to avoid using food as a treat or reward for polite/good behaviour – use positive verbal praise and hugs instead
- It is important not to pressure your child to eat – let them understand and express their own hunger and fullness cues
- Offer food around the same times each day. If children eat at regular mealtimes they are more likely to be hungry at that time of day
- Encourage them to eat more at mealtimes instead of more during snack times by serving small amounts at snack times. Your child needs small regular healthy snacks to keep their energy up, but if you provide too many snacks or a large amount, they can fill them up before a main meal
- Avoid offering your child an alternative if they don't eat a meal. Children can go through times when they have only a small appetite
- Make sure your child is not drinking too much milk – this fills them up and stops them being hungry for foods

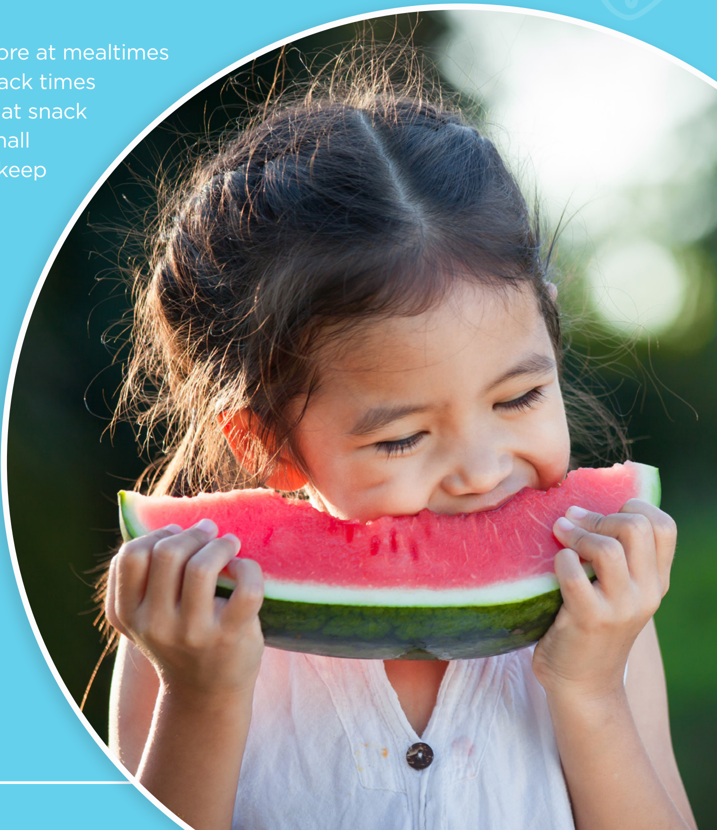

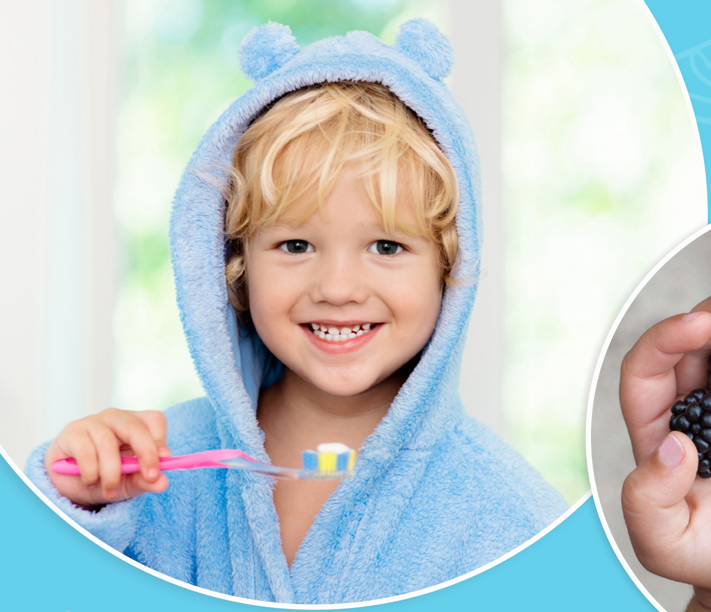

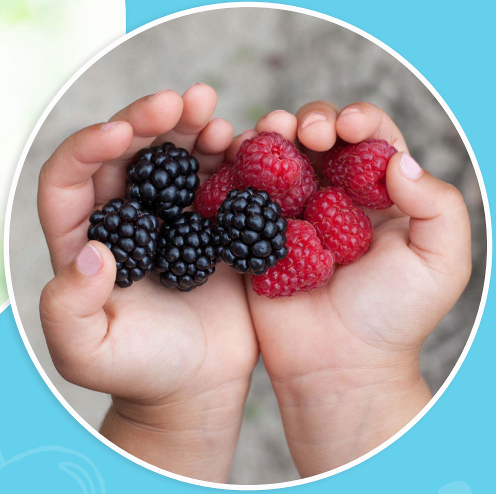

Looking after your child’s teeth

Children need a dental checkup every year to keep their teeth and gums healthy.

3 easy steps to keep your child’s teeth and gums healthy	
	**Offer healthy snacks and meals**<br>Foods that are high in salt, sugar or fat can cause tooth decay and are not good for their health
	**Offer water or milk from a cup**<br>Using a bottle or offering formula milk, flavoured milk, fruit juice, soft drinks or other sweetened drinks is not recommended. They can cause either tooth decay or can lead to low levels of certain nutrients that are important for their growth
	**Help your child brush their teeth morning and night**<br>Use low fluoride toothpaste

YOUR PRESCHOOLER'S PLAY

Preschoolers should spend at least 180 minutes a day in a variety of physical activities. This should include at least 60 minutes of energetic play such as running, jumping and kicking and throwing, spread throughout the day.

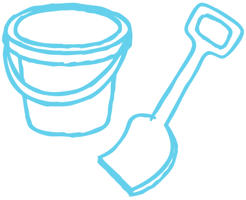

Some fun activities while at home:

- Play indoor hide and seek
- Family jigsaw puzzles
- Blowing and chasing bubbles
- Family yoga through YouTube
- Family dance-off competition
- Music concert with homemade instruments e.g. tapping sticks, drums, shakers or try body percussion
- Start a visual diary for the whole family where everyone draws using crayons, pencils, watercolours
- Indoor ball games using rolled up socks instead of balls
- Start a vegetable garden and watch it grow
- Zoom other family members and friends - your child will love to see their playfriends and family
- Cook or bake with your child – simple recipes where they can get involved
- Playdough or air drying clay
- Dominoes, and simple puzzles, card and board games
- Imaginative and pretend play – using props such as dress-ups, puppets or pots, pans and plastic containers and utensils
- Make a cubby house by using boxes, sheets, blankets, chairs and towels
- Starting a craft box is a fun and creative way of using paint, glue, cloth, pencils, crayons and wool to make pictures
- Download audiobooks for your child and/or the family to listen to all together

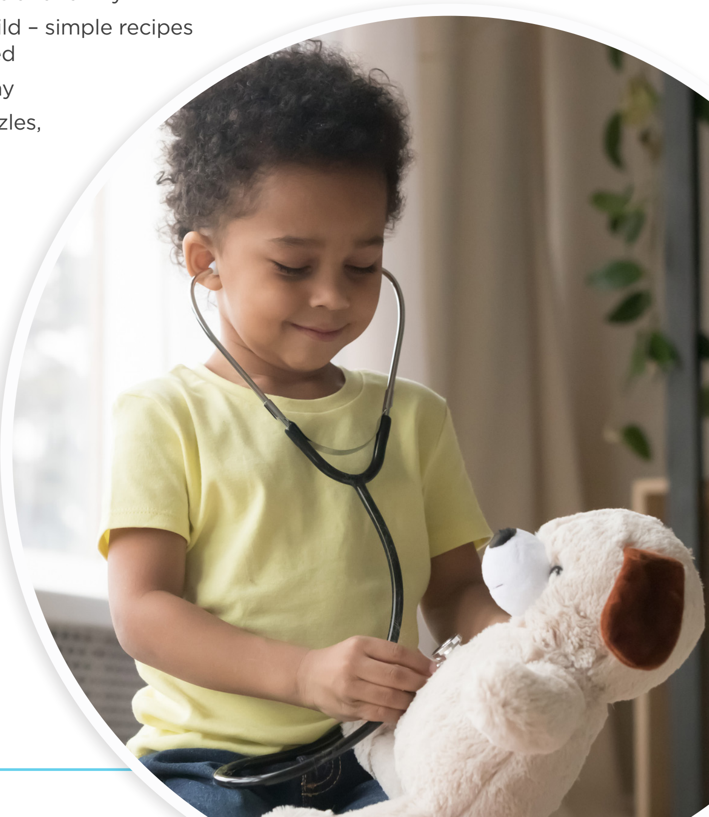

Fundamental Movement Skills

It is really important that you keep practising fundamental movement skills with your preschooler. These movements will help them play different games, sports and activities during preschool, primary school and high school.

Action	Step 1	Step 2	Step 3
**Stability skill**<br>**Hopping**	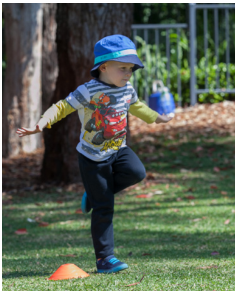<br>Stand on one leg and bend the opposite leg	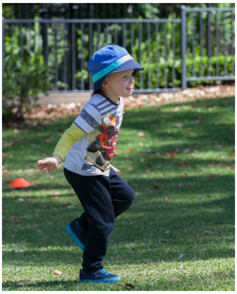<br>Spring off your foot	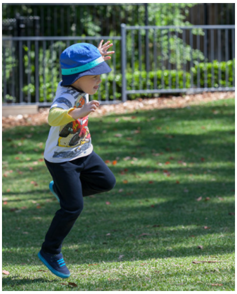<br>Use arm swing and leg swing to help you hop
**Locomotor skill**<br>**Galloping**	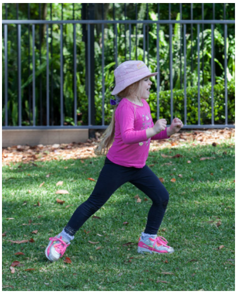<br>Step forward with one foot	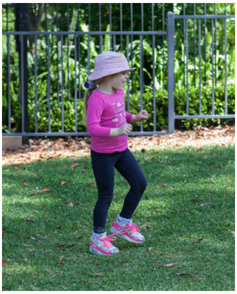<br>Second foot pushes the front foot in the air	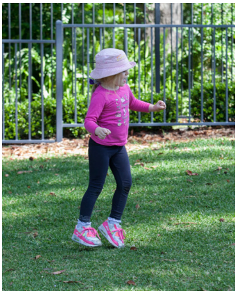<br>Toe to heal
**Manipulative skill**<br>**Overarm throw**	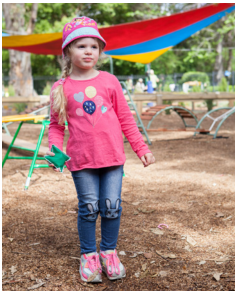<br>Point to where you want them to throw	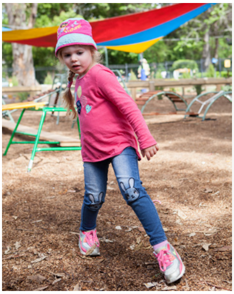<br>Swing arm back, step one foot forward and throw	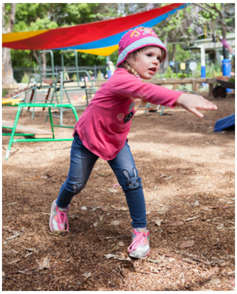<br>The throwing hand follows through across body towards hip.

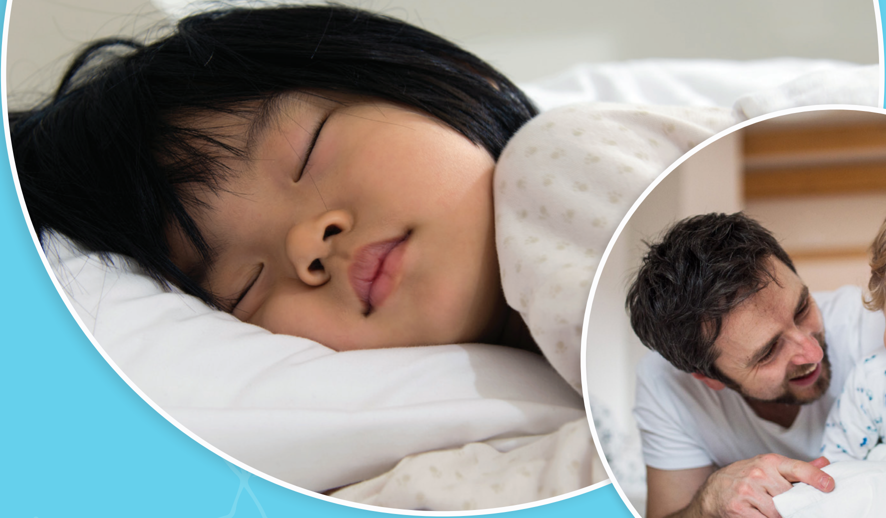

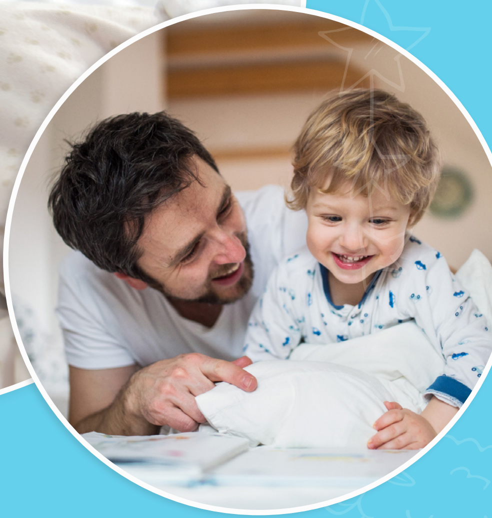

YOUR PRESCHOOLER'S SLEEP

Children aged 3 - 5 years need around 11 - 13 hours of sleep a night. Some might also have a day nap of about an hour. Sleep is important for your preschooler's health, growth and development. When children sleep well, they're more settled and happy during the day. Getting the right amount of sleep also strengthens your child's immune system and reduces the risk of infection and illness.

Getting up after bedtime

Your preschooler might go through stages of calling out from bed or getting up after you've said goodnight. The following are tips you can try:

- Before putting them into bed, help them wind down by telling them a bedtime story or reading a book
- Keep them away from any screens for at least 30 – 60 minutes before bedtime. When their eyes are exposed to bright light from a TV, computer, phone or tablet close to bedtime, it can make it harder for your child to settle
- Create a calming bedtime routine for them such as having a warm bath, brushing their teeth, sitting in bed and reading them a book
- Make sure their room is quiet and if you can, lower bright lights, use a lamp or night light
- Before leaving the room, check that your child has everything they need for sleep, like their favourite cuddly toy or blanket. Remind them to stay quietly in bed
- If your child gets out of bed, calmly ask them to go back to bed. Say that you're just in the other room. Repeat this firmly but in a calm tone over and over until they don't get up again

Sometimes your child may actually need something. If your child is scared of a monster under the bed, quickly check and tell them there are no monsters. They might settle after this. If your child is scared of the dark, think about using a night light.

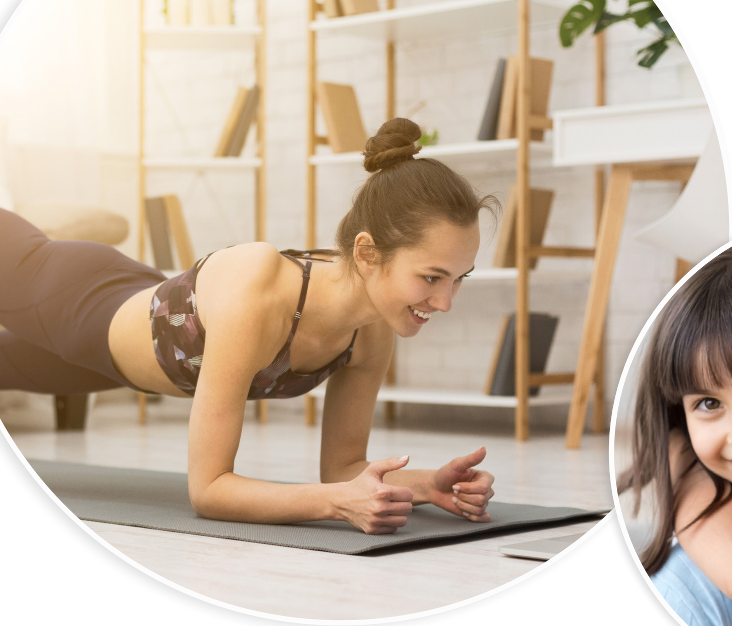

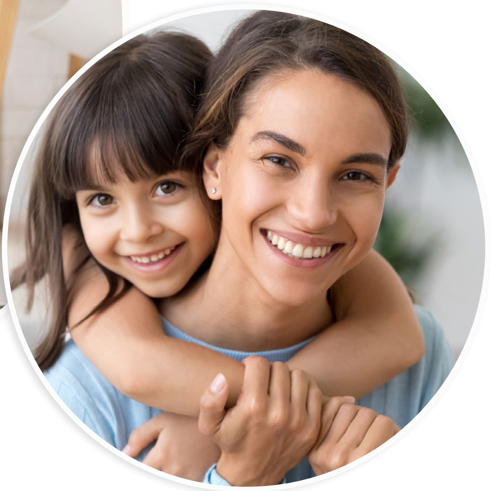

LOOKING AFTER YOURSELF

What are you currently doing to care for yourself?

The previous Healthy Beginnings booklets from birth to 3 years have tips on how to look after your overall wellbeing. You might know what is good for you but it is important that you remember to always have some time to put it into practice.

Try and find 10 – 20 minutes of time for yourself each day. Choose something you enjoy and that brings a smile to your face. By caring for yourself, it will help you stay positive in your day to day activities whether it is working, looking after your child or the family, or doing household chores.

Activities for your own self-care do not have to be costly. It might be as simple as sitting in the sunshine, talking to a friend, going for a jog, YouTube a yoga session, reading a book, listening to your favourite music or painting your nails.

Parenting can be exhausting and challenging, so if you ever feel like you need support or help try and reach out to your friends or family. If you are not enjoying your usual activities, feeling overwhelmed or experiencing a persistent low mood, talk to your CFHN, GP or one of the services below.

SERVICES AVAILABLE

beyondblue 1300 224 636

Pregnancy, Birth & Baby 1800 882 436

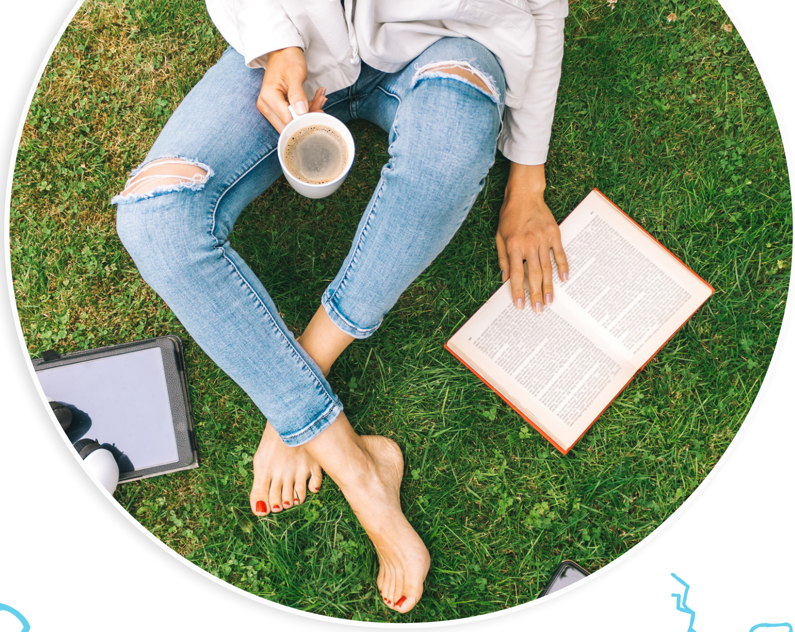

WHAT TO CONSIDER FOR YOUR CHILD'S HEALTH 3 - 5 YEARS

Keep me healthy, give me fruit and vegetables every day

Water in a cup is best for me and my teeth

Keep me active, I don't need screen time for play

I'm a preschooler now, not a baby, I can sleep at night without any milk

Reward me with cuddles and praise, food rewards are unhelpful for me

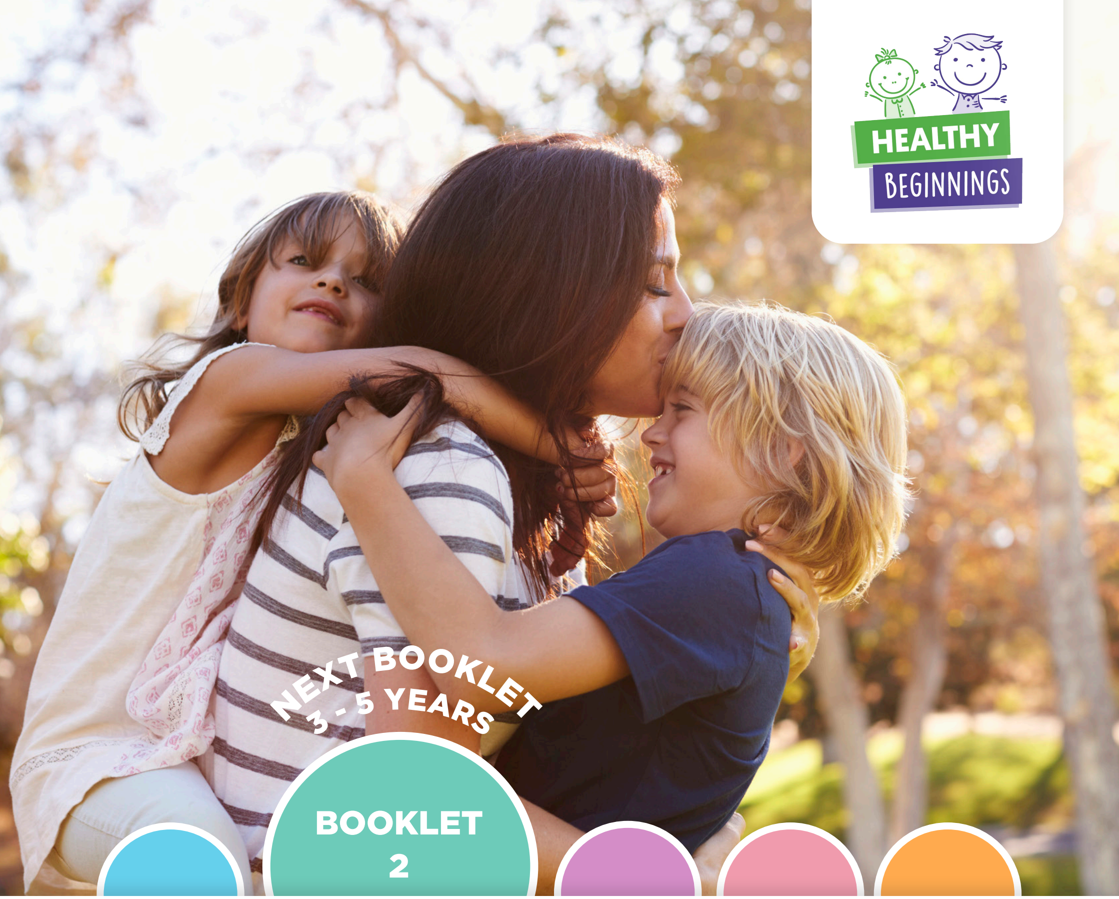

CONTACT

HB

Healthy Beginnings
SLHD-HealthyBeginnings@health.nsw.gov.au
www.healthybeginnings.net.au
02 9515 9055

HD

Health Direct Australia
www.healthdirect.org.au
1800 022 222

Acknowledgments The Healthy Beginnings program was funded by the NSW Health Translational Research Grant Scheme 2016 (ID number: TRGS 200) and NHMRC Partnership Project 2019 (#APP1169823). We sincerely thank participating partners of the project from the Sydney, South Eastern Sydney, South Western Sydney and Southern NSW Local Health Districts, and members of the advisory committee and project management team. This resource has been prepared with the guidance of health professionals. The information for this resource was obtained from NSW Health sources, National Health and Medical Research Council, Australian Breastfeeding Association and Raising Children Network. The information in this resource is for general guidance only and should not be a substitute for professional advice.

NSW GOVERNMENT

July 2020
