# Supplementary material for: Effectiveness of a telephone-based randomised clinical trial targeting obesity risk of preschool-aged children: An extension study during the COVID-19 pandemic
Source: Int J Obes (Lond). 2025 Aug 14;49(11):2241–8. doi: 10.1038/s41366-025-01869-4 (PMC12583190; doi:10.1038/s41366-025-01869-4)
Supplement: Supplementary file 3 — Supplementary Document 1_3-4 years Booklet 2 [file 41366_2025_1869_MOESM3_ESM.pdf]

THE PRESCHOOL YEARS
3-5 YEARS

Booklet 2

Supporting your family during your child's preschool years

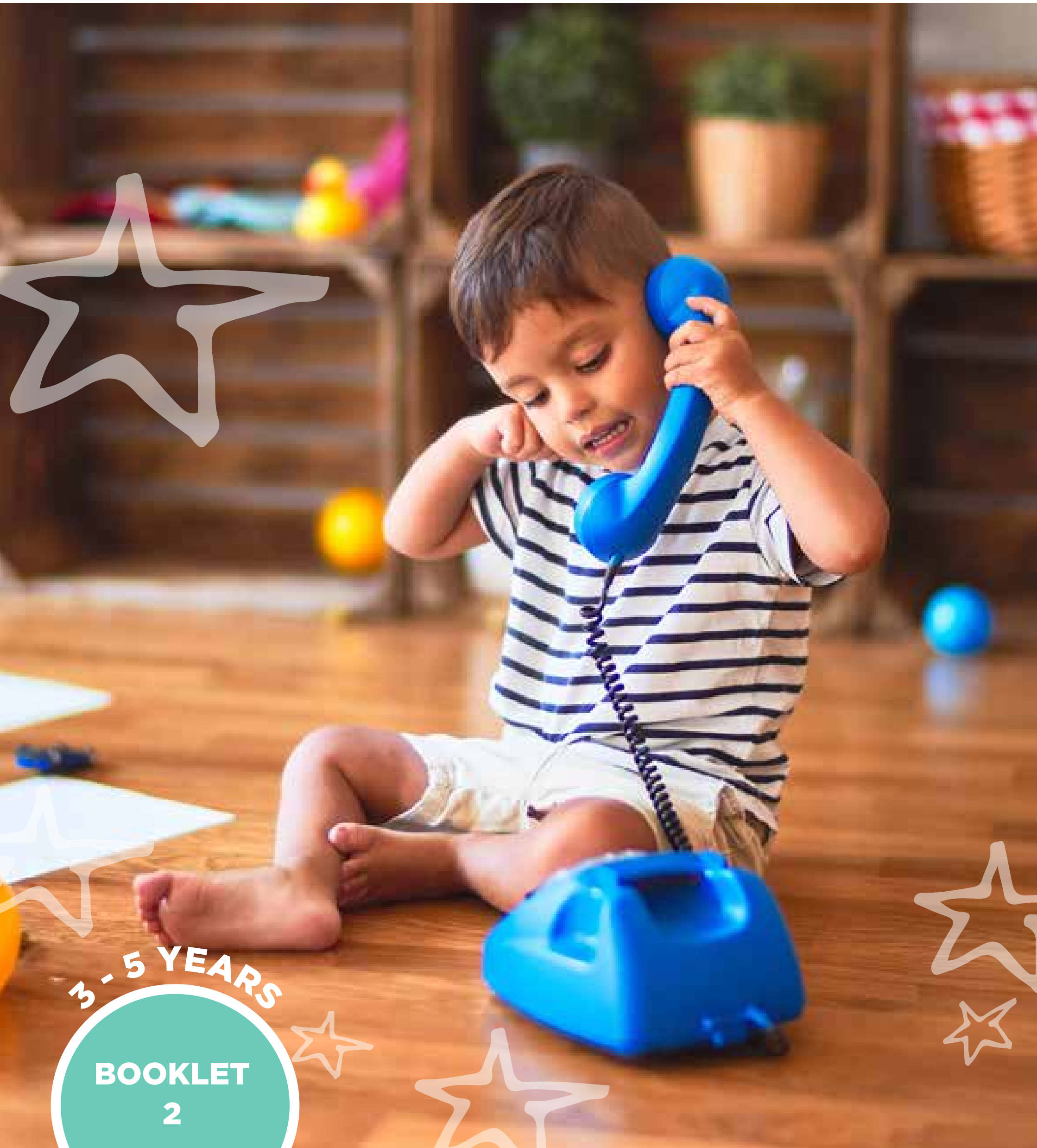

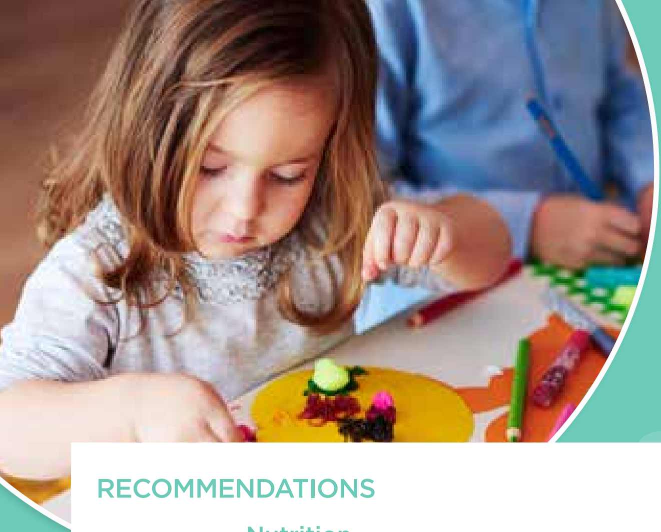

YOUR CHILD AT 3 - 5 YEARS

Healthy eating and active play are important for your preschooler.

This booklet provides information on your preschooler's health and tips for parents' health too.

RECOMMENDATIONS

Nutrition

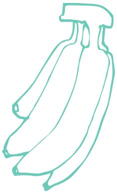

- It is best for your child to drink from a free-flow cup or an open cup and to stop all bottle feeds
- Drinks can include:
  - o water
  - o reduced fat cow's milk
  - o calcium enriched soy, rice or oat milk can be used, but it is important to plan this with a health professional
- Preschoolers need lots of different foods every day. This includes aiming for 2.5 serves of vegetables and 1 serve of fruit
- Keep breastfeeding, if you and your child are happy to keep going. For support, contact Australian Breastfeeding Association **www.breastfeeding.asn.au**

Active play

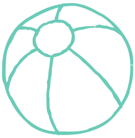

- **Lots of exercise and play:** Make sure your child spends at least 3 hours doing lots of different activities, every day
- **Staying active:** It's good for children to be active! Try to make sure they aren't still for longer than one hour at a time, unless they're sleeping
- **Screen time:** Between 3 - 5 years, children should be limited to less than 1 hour of screen time in a day. This can include watching or playing an educational program or using an app with an adult. Spending less time on the screen is better
- **Sleep:** 11 - 14 hours of good quality sleep every day. This can include night sleeps and naps

Immunisation

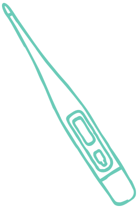

- Your child is due a 4 year Personal Health Record (blue book) developmental check so book an appointment with your Child and Family Health Nurse (CFHN) or GP
- Your child's next immunisation is at 4 years Check **www.nsw.health.gov/immunisations** for more details
- Other immunisations are available - please discuss with your GP
- Your child is also due a yearly dental check – either with your family dentist or your local Community Health Dental Clinic

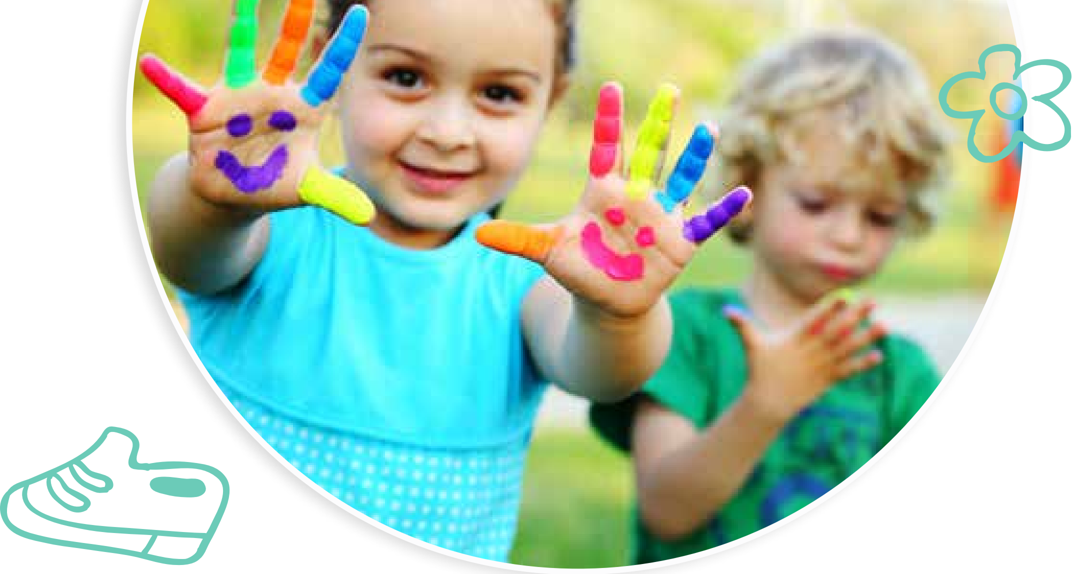

WHAT TO EXPECT AS A PARENT

From 3 – 5 years preschoolers will continue to develop physically and emotionally.

Your child will become more interested in playing and making new friends. They will explore their feelings and learn how to share through play and exploration. During this time, they will develop their understanding of concepts such as 'mine', 'his/hers' and 'theirs' while playing in small groups. They start to become curious and enjoy using their imagination for pretend and creative play. They might also ask you lots of questions such as "why" and "who".

Self-regulation will begin to develop as they start to understand and manage their behaviour, feelings and reactions to things happening around them. This includes calming their emotions like frustration or excitement, re-focusing on learning new tasks, learning emotional cues and behaviours that help their friendships develop.

Preschoolers love praise and hearing positive words and gestures (like a high five or clapping) from their parents after good behaviour. Your child will feel nurtured and confident after receiving praise.

Your child will start to become more independent and are able to dress themselves, enjoy family meal times and feed themselves. They may need some assistance with toilet time, and reminders as well as help to wipe their bottom and wash their hands. It is also best to supervise teeth brushing twice a day.

If you are concerned about your child's behaviour, please speak with your CFHN or GP. For information on parenting courses to support your child's social and emotional development visit www.resourcingparents.nsw.gov.au

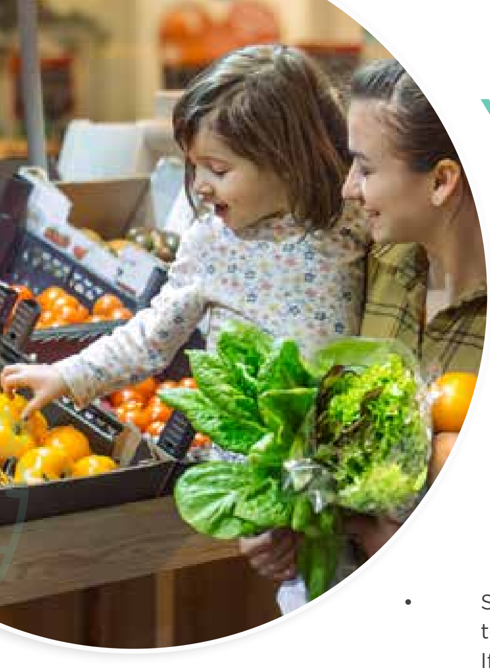

YOUR PRESCHOOLER'S NUTRITION

Grocery shopping with children can be hard but it can also be a positive experience. Use this time to connect and to educate them about healthy food choices.

Tips to help you shop with your preschooler:

- Prepare a shopping list and stick to it! Try not to get distracted by sales on food that is high in sugar, fat or salt, like biscuits, chocolate or chips
- Set expectations with your child before your trip to the shops about what you are going to buy. If they behave while shopping, reward them with a trip to the playground. Take healthy snacks and water to drink
- Eat a healthy snack before shopping so you and your child are not hungry and end up buying unhealthy food
- Fill your trolley with mostly seasonal fresh produce like fruit and vegetables
- Try to avoid the aisle with lollies, soft drinks, biscuits and chips
- Buy frozen or canned fruit and vegetables to save time and money
- Make grocery shopping fun and educational. You can ask them to choose fruit and count as they place them in the bag
- Ask them to help you pack the shopping away in the fridge when you get home
- Plan a regular shop, once a week. Having healthy family food ready in the house makes it easier for your family to put together a healthy snack or meal
- Plan your weekly meals with your child and let them choose a meal they can help you prepare. See https://www.makehealthynormal.nsw.gov.au/food/weekly-menu-plan

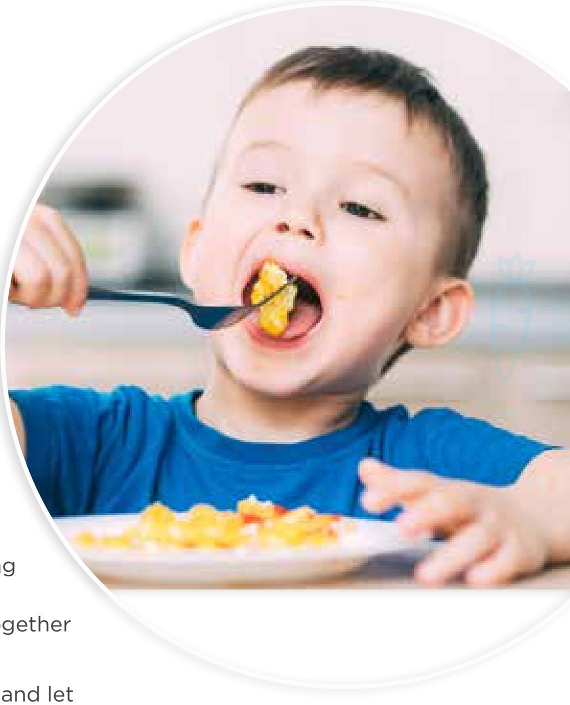

Drinks

Encourage water as the main drink

- Have water in the fridge that your child can access easily
- Serve water with all snacks and family meals
- Add a refillable water bottle to your preschooler's lunch box
- Take water bottles when your child plays outdoors
- They don't need sports drinks as they are very high in sugar
- Show your child you enjoy water to drink
- Add some berries or sliced oranges and ice to a jug of water
- Don't have sweet drinks in the house - these can be a "special " or a "sometimes drink"

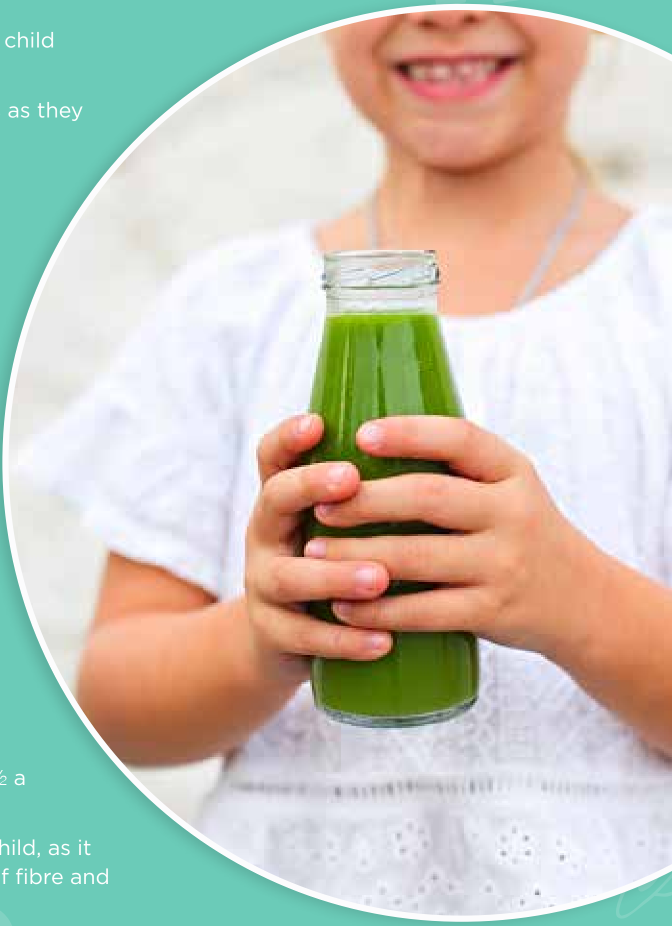

Juice and soft drinks

Your preschooler may start to notice and ask for drinks like soft drink and flavoured milk. Remind them that they are "special" and not for every day. Water is the healthiest option.

- Limit juice intake. The most a child should drink per day is ½ a cup (125ml)
- Fresh fruit is better for your child, as it keeps them full and has lots of fibre and vitamins

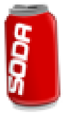

up to 16 tsp of sugar (soft drink)

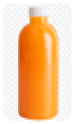

up to 12 tsp of sugar (juice)

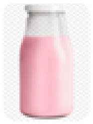

up to 14 tsp sugar (flavoured milk)

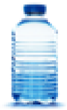

zero sugar (water)

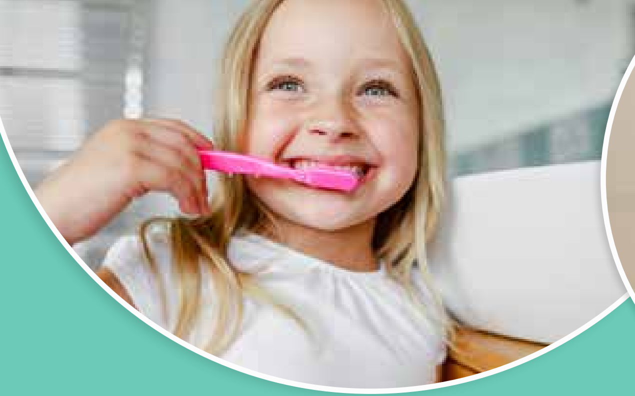

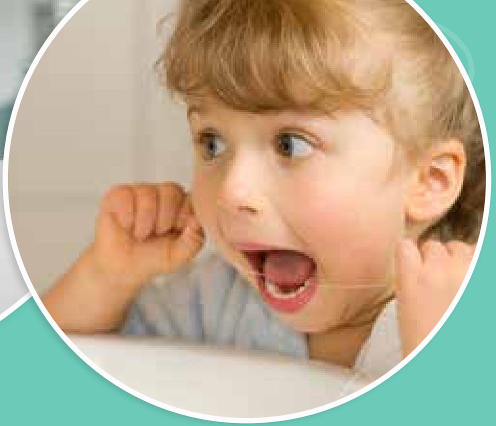

Looking after your child's teeth

Children need a dental checkup every year to keep their teeth and gums healthy.

3 easy steps to keep your child's teeth and gums healthy	
	**Offer healthy snacks and meals**<br>Foods that are high in salt, sugar or fat can cause tooth decay and are not good for their health
	**Offer water or milk from a cup**<br>Using a bottle or offering formula milk, flavoured milk, fruit juice, soft drinks or other sweetened drinks is not recommended. They can cause either tooth decay or can lead to low levels of certain nutrients that are important for their growth. Water is great to rinse their mouth with after eating.
	**Help your child brush their teeth morning and night with low fluoride toothpaste**<br>Support them with cleaning hard to reach areas
	**Your child should also regularly floss any teeth that touch each other**<br>Book in to see the dentist if you notice:<br>• Teeth are brown, black or stained yellow<br>• Thumb sucking, grinding or injuries to teeth<br>Remember they also need a yearly dental check

YOUR PRESCHOOLER'S PLAY - Aim for 3 hours a day

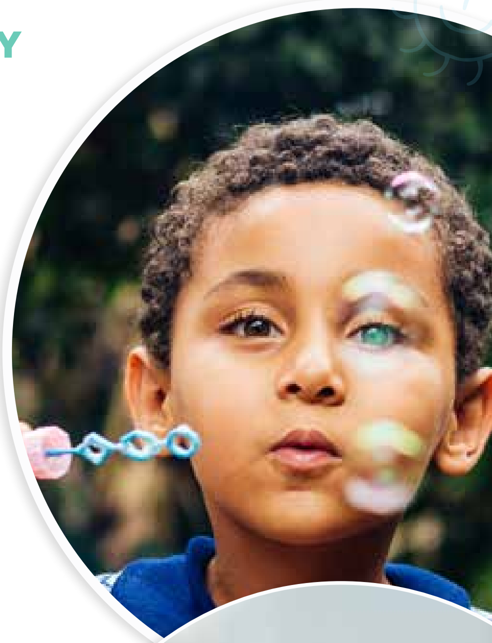

Preschoolers should spend at least 180 minutes (3 hours) a day being physically active. Spread throughout the day, this should include at least 60 minutes (1 hour) of energetic play such as running, riding a bike, climbing on play equipment, kicking and throwing.

Less energetic activities like putting toys away, helping with everyday household tasks, and walking, all count as being active.

Keep introducing activities to your daily routine to develop healthy habits for life. For example, go for a quick walk around the block after breakfast or have a dance to a few fun songs at home.

Outdoor play is important for your child's physical and emotional development and wellbeing. They have more freedom to make noise, a mess and move around on different surfaces. Playing in the outdoors helps to promote a calming and happy mood, build self-esteem and confidence, and develop balance, coordination and strength. Energetic play outside can help them sleep better too.

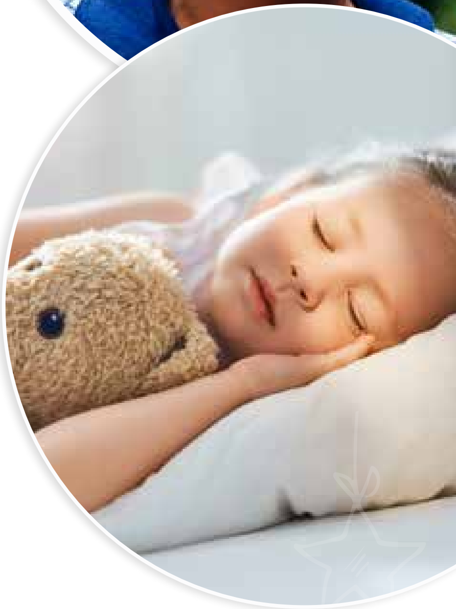

YOUR PRESCHOOLER'S SLEEP

Set a bed time for your child (around 7 - 8pm) so that they can get enough hours of sleep every night. Sleep is important for your child's mood, growth, learning and development.

Aim for 11 - 14 hours of good quality sleep every day, including night sleeps and day naps.

Sample nighttime routine:

- Dinner between 4.30 - 6.00pm
- Warm bath
- Aim for around 20 minutes of quiet and positive time for cuddles, calm songs or storytelling
- No screens for at least 1 hour before bed time
- If you would like support with changing your child's bedtime routine see your CFHN or call Karitane 1300 227 464 or Tresillian 1300 272 736, or online chat services

TOILETING YOUR PRESCHOOLER

Encouraging healthy toileting habits

Children are usually toilet trained by about three years of age. Even after this, occasional accidents can happen like soiling (poo) in your child's underwear.

If regular accidents occur or there are ongoing issues with toilet training when your child is 3 - 5 years old, then further help is required. See your GP and your CFHN for a medical assessment.

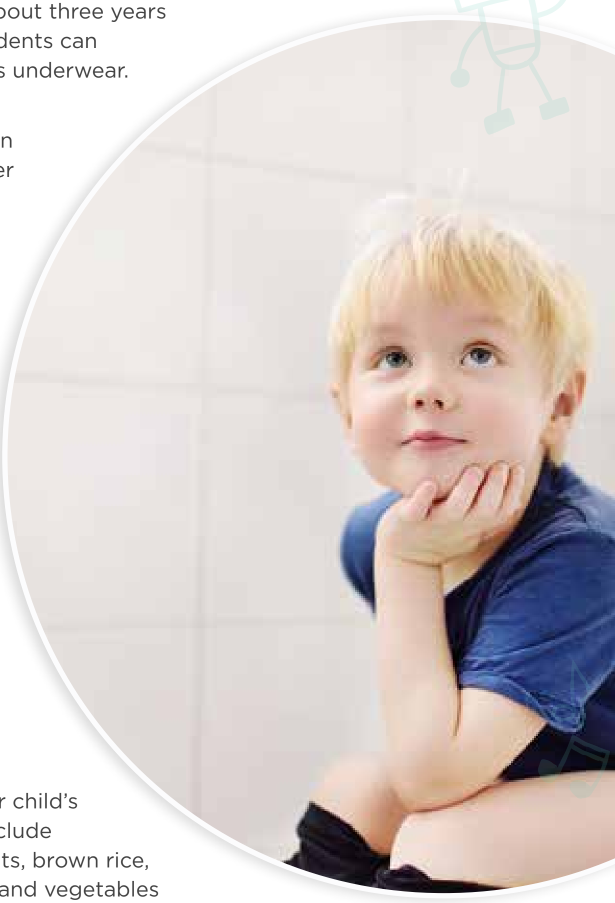

Encourage your child to sit on the toilet for 3 - 5 minutes: in the mornings, before or after meals and snacks, and before bed. Use a timer.

Most children have a bowel movement at least once a day or every 2 - 3 days.

Some may go up to 3 times a day. This is all normal as long as the poo is soft and easy to pass.

Constipation is when your child has dry, firm, and hard to pass bowel movements.

Tips to prevent constipation:

- Ensure there is enough fibre in your child's diet. Foods that are high in fibre include wholegrain breads and cereals – oats, brown rice, brown pasta, legumes, beans, fruit and vegetables
- Offer your child plenty of water
- Encourage your child to do a physical activity, which helps with bowel movement regulation
- Encourage your child to sit on the toilet about 20 minutes after at least one meal a day; after breakfast is often a good time. Also encourage them to go when they feel the urge or need to go and not to 'hold on'
- Your child may need a footstool to encourage good positioning on the toilet, which makes it easier to pass a poo
- Show your child picture books about going to the toilet, make it normal and fun

Remember:

- It is very important to use positive words and remain calm if your child has an accident. Encourage their attempts and praise your child for sitting on the toilet and trying
- Talk to your GP if you are worried about regular accidents, constipation or soiling
    - o Make a diary of your child's bowel movements and accidents for a couple weeks. Take this to the GP appointment
    - o Your CFHN, GP or dietitian may also assess the child's dietary habits

VCRC

THE BRISTOL STOOL FORM SCALE (for children)

Choose your Poo!

type 1	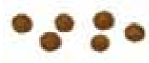	looks like: **rabbit droppings** Separate hard lumps, like nuts (hard to pass)
type 2	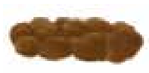	looks like: **bunch of grapes** Sausage-shaped but lumpy
type 3	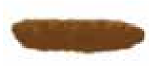	looks like: **corn on the cob** Like a sausage, but with cracks on the surface
type 4	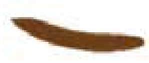	looks like: **sausage** Like a sausage or snake, smooth and soft
type 5	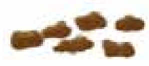	looks like: **chicken nuggets** Soft blobs with clear-cut edges (passed easily)
type 6	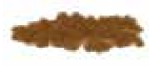	looks like: **porridge** Soft blobs with clear-cut edges (passed easily)
type 7	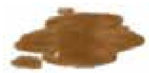	looks like: **gravy** Watery, no solid pieces ENTIRELY LIQUID

The concept by Professor DCA Candy and Emma Davey, based on the Bristol Stool Form Scale produced by Dr KW Heaton, Reader in Medicine at the University of Bristol. © 2005 Norgine Limited manufacturer of MOVICOL®-Half

Reproduced with kind permission from Dr KW Heaton, formerly Reader in Medicine at the University of Bristol, ©2000-2020, Norgine group of companies.

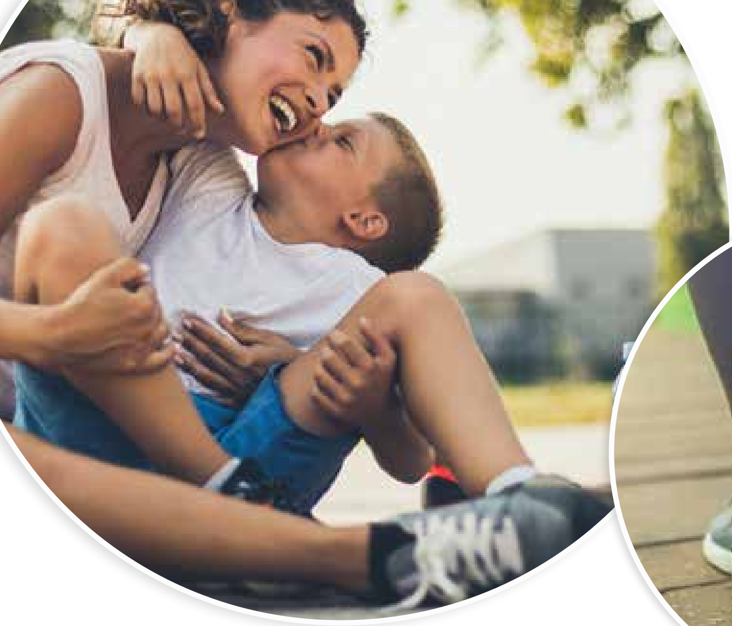

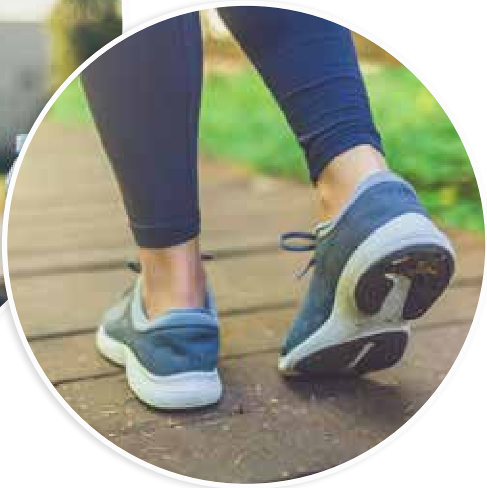

LOOKING AFTER YOURSELF

As a parent it is common that you put the needs of your family before yours. There are many responsibilities that you may be juggling like parenting one or more children, nurturing personal relationships, employment (including career progression), studying, extended family commitments, community responsibilities, household tasks - just to mention a few.

In all our Healthy Beginnings booklets we encourage you to take time for self-care to rest, reset and rejuvenate so you can avoid and reduce the symptoms of stress and anxiety.

Research suggests that connection with others (online, in person), mindfulness, doing activities you enjoy like singing, drawing, reading and walking all help support mental wellbeing. Self-care is beneficial to you personally and those that you care for, giving you optimal energy and awareness. Aiming for balance in your daily routine will help you be more productive and resilient to daily stressors in your life.

What are you currently doing to care for yourself?

It is important to have your physical, emotional, and dental health regularly monitored too.

Are you due for a regular health check? It is easy to forget that your own health needs monitoring. Routine blood pressure and blood tests, breast, cervical and skin cancer screening as well as dental check-ups should all be carried out regularly.

Book in a time to see your GP to talk about your health.

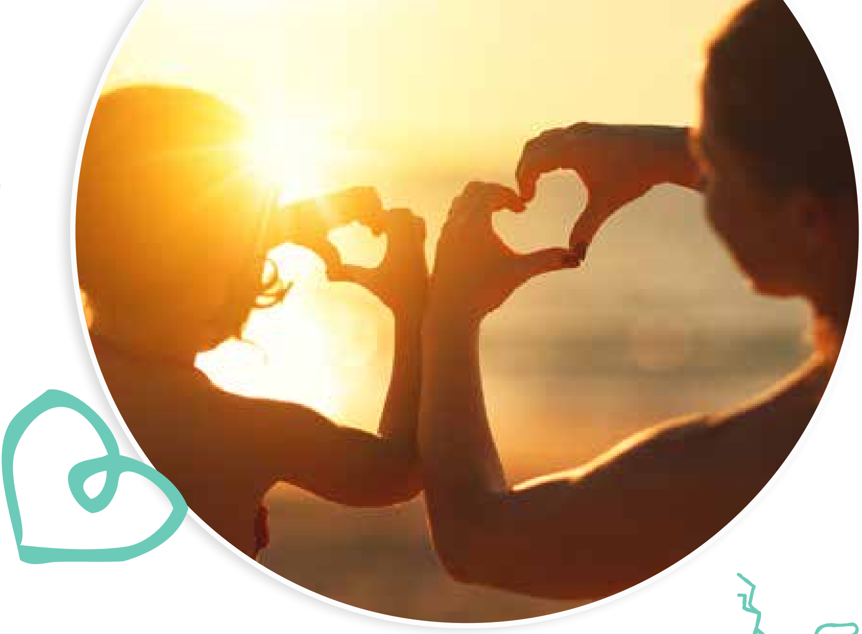

WHAT TO CONSIDER FOR YOUR CHILD'S HEALTH 3 - 5 YEARS

- Keep me healthy, give me fruit and vegetables every day
- Water in a cup is best for me and my teeth
- Keep me active, I don't need screen time for play
- I'm a preschooler, not a baby, I can sleep at night without any milk
- Reward me with cuddles and praise, food rewards are unhelpful for me

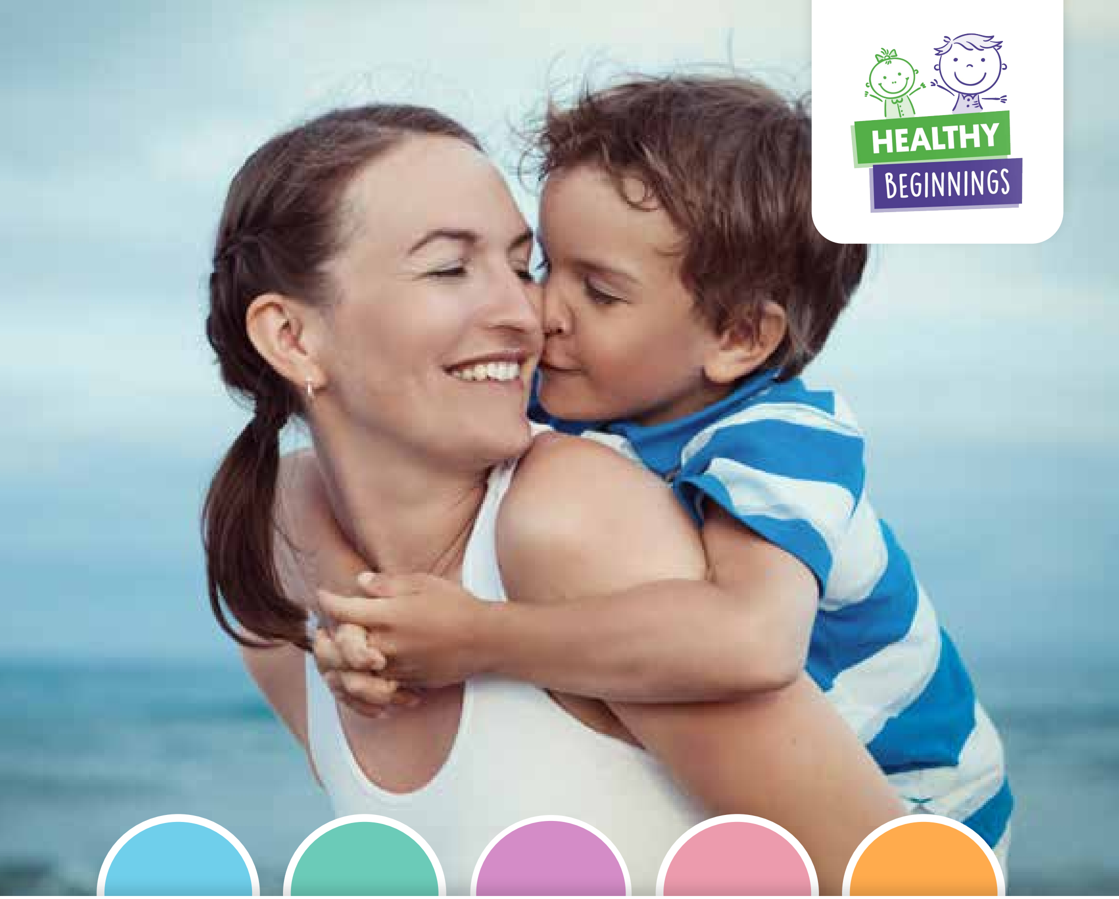

CONTACT

HB

Healthy Beginnings
SLHD-HealthyBeginnings@health.nsw.gov.au
www.healthybeginnings.net.au
02 9515 9055

HD

Health Direct Australia
www.healthdirect.org.au
1800 022 222

Acknowledgments The Healthy Beginnings program was funded by the NSW Health Translational Research Grant Scheme 2016 (ID number: TRGS 200) and NHMRC Partnership Project 2019 (#APP1169823). We sincerely thank participating partners of the project from the Sydney, South Eastern Sydney, South Western Sydney and Southern NSW Local Health Districts, and members of the advisory committee and project management team. This resource has been prepared with the guidance of health professionals. The information for this resource was obtained from NSW Health sources, National Health and Medical Research Council, Australian Breastfeeding Association and Raising Children Network. The information in this resource is for general guidance only and should not be a substitute for professional advice.

NSW
GOVERNMENT

December 2020
