# Supplementary material for: Effectiveness of a telephone-based randomised clinical trial targeting obesity risk of preschool-aged children: An extension study during the COVID-19 pandemic
Source: Int J Obes (Lond). 2025 Aug 14;49(11):2241–8. doi: 10.1038/s41366-025-01869-4 (PMC12583190; doi:10.1038/s41366-025-01869-4)
Supplement: Supplementary file 5 — Supplementary Document 2_3-4 years_Tel 2 [file 41366_2025_1869_MOESM5_ESM.docx]

**Tingle 14**

Please complete the survey below.

Name=[demographics_arm_1][mother_first_name] [demographics_arm_1][mothers_surname]

Child’s name=[demographics_arm_1][baby_name]

Child’s age (in months)=[stage_14_arm_1][child_age_t14]

Thank you!

Please complete the survey below.

Study 1 group=[demographics_arm_1][s1_group]

Study 2 group=[demographics_arm_1][s2_group]

Name = [demographics_arm_1][mothers_first_name] [demographics_arm_1][mothers_surname]

Pre-schooler's name = [demographics_arm_1][baby_name]

Pre-schooler's gender=[demographics_arm_1][baby_sex] Thank you!

Child age

This participant has withdrawn from the study. Do not complete any further forms.

Date

Time

User

Your Name

- Wendy Smith
- Annmaree Lavery
- Trisha Cant
- Christine Phillis
- Nada Caroll
- Other/Admin

other specify

"Hi, [demographics_arm_1][mothers_first_name],

My name is . I am a Specialist Child and Family Health Nurse from the Healthy Beginnings Program. I am calling you today as you are part of the Healthy Beginnings Program, and you are now due your next telephone support call from our service.

Do you have time to talk with me now, or would you like to arrange another time or day that suits you?

I have up to 30 minutes available to talk with you now, so the length of our call will depend on how much information you would like at this stage.

I can offer you health information and support, and can discuss your and your pre-schooler's health. I am happy to answer any questions that you may have at this time, and will be asking you questions as part of the program. We will also be setting some health goals together that will be emailed to you at the end of the call.

Just to remind you that the call may be monitored, and that all information is kept confidential, except where there are safety concerns for you or your child. I may need to share your information to help keep you both safe.

If there are any questions you don't feel comfortable answering, then please don't feel you have to answer them.

**Topics to be covered**

- see phone script
- introduce yourself
- mention anonymity
- mention confidentiality

(Select boxes as you discuss)

So are there any recent changes in your life that may affect how we talk today?

(for example any changes in your or your pre-schoolers health / changes in your family situation / or have you moved house recently, etc.)

- Yes
- No

If yes, please detail

Have you received the Healthy Beginnings 3 – 4 year booklet 2?

- Yes
- No

Have you had a chance to read the booklet? Have you had a chance to read the booklet?

Yes No

(If only read partially, tick Yes)

- Yes
- No

Do you have any feedback on the booklet ?

What did you like / dislike about it?

Are you receiving the SMS messages from Healthy Beginnings?

- Yes
- No

Do you find them useful ?

- Yes
- No

Any comments about SMS:

Because you will get an email at the end of this call with the goals we will set together today, can you please confirm your email address?

Email address=[demographics_arm_1][email]

Today we will talk about:

- Healthy Eating for you and your pre-schooler
- pre-schooler physical activity and active play
- Sleep
- Looking after yourself

(make sure to check off as you discuss) (Select boxes as you discuss)

Any additional comments?

How are things going with [demographics_arm_1][baby_name]?

Do you have any concerns with [demographics_arm_1][baby_name] development?

Development information needed?

- Yes
- No


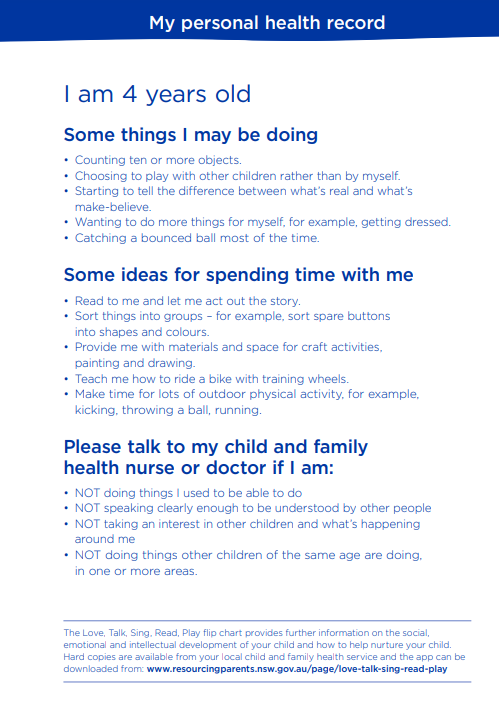


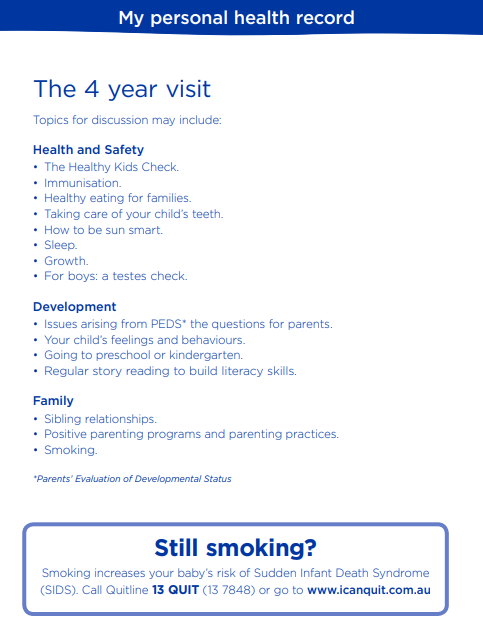


Do you know there is a 4 year PHR Blue Book developmental check that you can have completed at your Child and Family Health Centre, or with your GP. Your child has immunisations due at 4 years of age.

Check out [www.nsw.health.gov/immunisations](http://www.nsw.health.gov/immunisations) for more details

Do you have any concerns about your child’s emotional development or behaviour?

⧠ Yes

⧠ No

Coments:

_______________________________________________________________________

_________________________________________________________________________________________________________________

Drop down

WHAT TO EXPECT AS A PARENT

From 3 – 5 years preschoolers will continue to develop physically and emotionally.

Your child will become more interested in playing and making new friends. They will

explore their feelings and learn how to share through play and exploration. During

this time, they will develop their understanding of concepts such as ‘mine’, ‘his/hers’

and ‘theirs’ while playing in small groups. They start to become curious and enjoy

using their imagination for pretend and creative play. They might also ask you lots of

questions such as “why” and “who”.

Your child will begin to develop self-regulation with your support: this means they will start to understand and manage their behaviour, feelings and reactions to things happening around them.

This includes managing their emotions like frustration or excitement, refocusing on

new tasks, calming themselves down after being upset, and learning emotional cues and

behaviours that help their friendship development.

Preschoolers love praise and hearing positive words and gestures (like a high five or

clapping) from their parents after good behaviour. Your child will feel nurtured and

confident after receiving praise.

Your child will start to become more independent. They are able to dress themselves,

enjoy family meal times and feed themselves. They may still need some assistance with

toilet time, like reminders and support to wipe their bottom and wash their hands. Dentists recommend twice a day.

If you continue to be concerned about your child’s behaviour and you are not seeing the beginnings of them being able to regulate their emotions, please speak with your Child and Family Health Nurse or GP. Please see [www.resourcingparents.nsw.gov.au](http://www.resourcingparents.nsw.gov.au) for information on parenting courses to support your child’s social and emotional development.

Key Messages

- Keep me healthy, give me fruit and vegetables every day
- Water in a cup is best for me and my teeth
- Keep me active, I don't need screen time for play
- I'm a pre-schooler now, not a baby, I can sleep at night without any milk
- Reward me with cuddles and praise, food rewards are unhelpful for me

**KEY AREA 1 –Pre-schooler Nutrition**

T11 goal for [demographics_arm_1][baby_name]'s food & feeding=[stage_11_arm_1][new_fgoal_t11]

T12 goal for [demographics_arm_1][baby_name]'s food & feeding=[stage_12_arm_1][cnutri_goal_t12]

We are now going to talk about your pre-schooler’s nutrition and pre-schooler daily eating.

How is [demographics_arm_1][baby_name] going with eating fruit and vegetables every day?

Comment

The current, daily recommendations for pre-schooler's are:

FRUIT: 1 / day recommended, 1 serve = 1 medium piece or two small pieces of fruit or 1 cup of diced pieces VEGETABLES: 2-3 / day recommended, 1 serve = ½ cup cooked vegetables or 1 cup of salad vegetables What to feed and how much food

At 3 – 4 years your pre-schooler will mainly be eating the same food as the rest of the family. This might include eating 3 healthy meals a day and 2 healthy snacks in between. At this age, pre-schooler’s are able to express what they like and dislike whether they are hungry or full. That is why it is important that parents provide a variety of healthy food and children can decide how much to eat.

Some children prefer eating fruit over vegetables. This is usually because fruit is sweeter.

It is important to encourage vegetable eating by offering a variety of different coloured vegetables, every day.

Vegetables can be raw or cooked, fresh or frozen, including green, red, yellow and orange ones e.g. mashed sweet potato, boiled broccoli , carrots with a yoghurt dip or a pasta sauce made with tomatoes, grated zucchini / carrot and mushrooms.

Suggestion daily dietary guidelines for pre-schoolers at 3-4 years


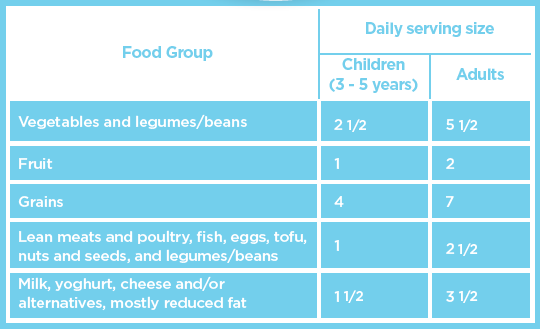


Do you have any concerns about your child’s eating and drinking?

- Yes
- No

Do you think they are getting enough calcium rich foods every day?

- Yes
- No


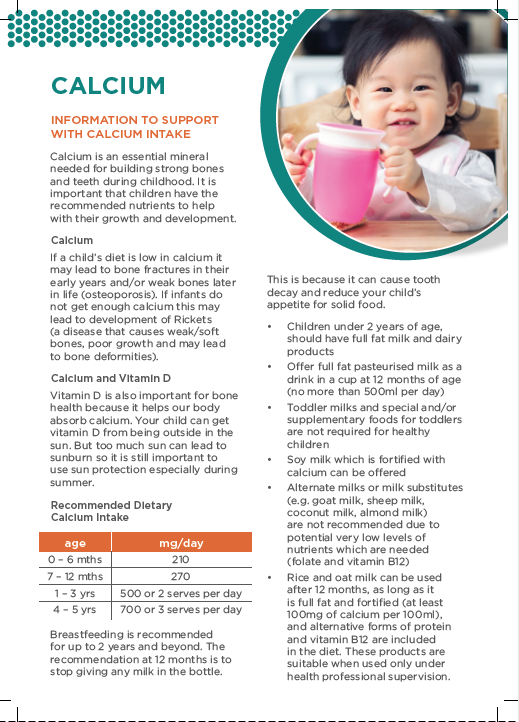


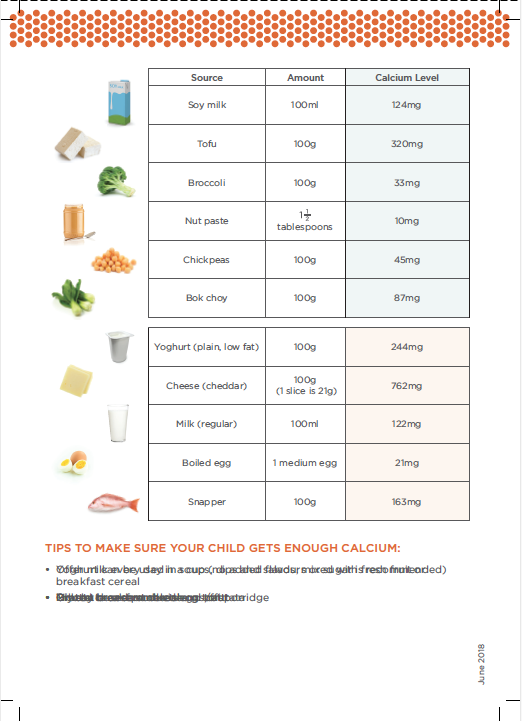


Do you think they have iron-rich foods every day?

- Yes
- No


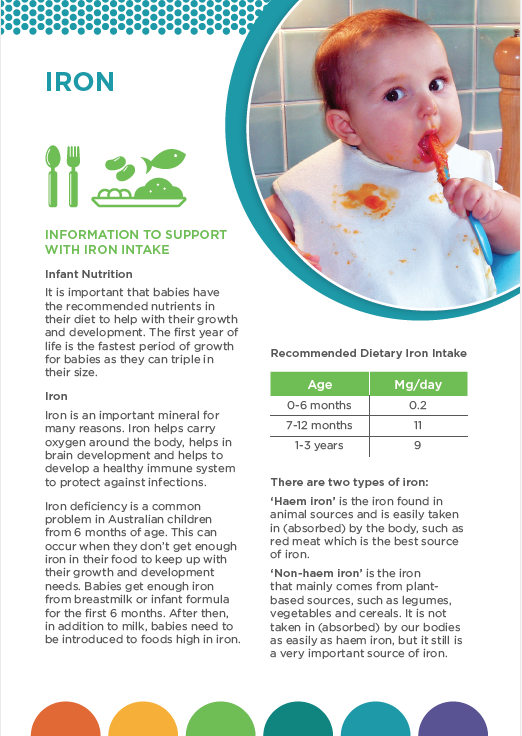


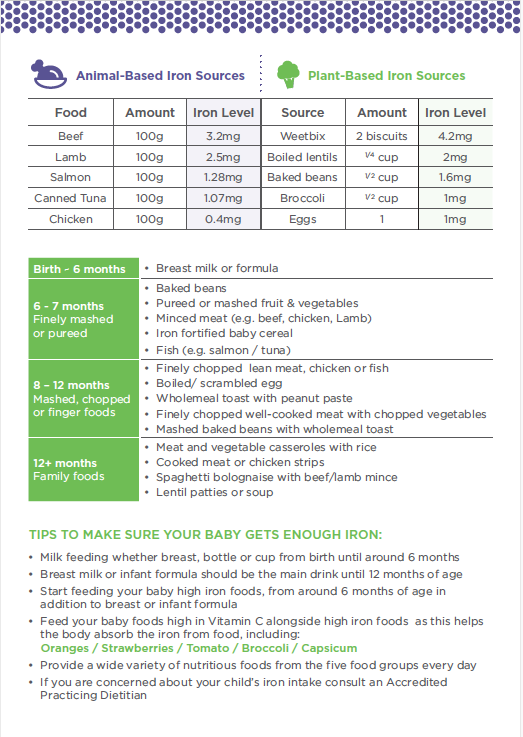


Are you eating together as a family?

- Yes
- No

pre-schoolerAre you eating together as a

How often?

- The serving size (the amount of food) children at 3 – 4 years should have is about half of an adults’ serving. So the portion size on their plate at each meal will also be about half the size of your plate. The recommendations are a general guideline based on age and activity levels.
- Family mealtimes are important as your child pre-schooler is learning good eating habits from you, are more likely to try new foods if the family is eating them, and eating together is a great time to connect and talk with each other. It is a social time - where they are learning to take turns in talking and listening - social skills for life! Having a screen on - TV / Ipad / Mobile phone, can stop this happening and stop them recognising their fullness and hunger cues. Eating together as a family - with screens turned off - also allows your child to see you eat, can decrease any food fussiness or fussy eating, and allows you to be their food role-model, as they are watching you eat and copying you.
- Your pre-schooler will copy your eating habits - so it is important that they see you eating fruit and vegetables - and a wide variety of healthy foods.

Family mealtimes and eating healthy

- Most importantly - Try to eat meals together as a family and as often as possible - choose at least 1 meal a day to eat together, talk and enjoy your time in a relaxed way. If your pre-schooler is rushed or pressured to eat this can make them feel stressed and release hormones that makes them lose their appetite. This can make meal times unhappy and stressful, rather than relaxed and pleasant
- It can take around 20 minutes from when your preschooler pre-schooler begins to eat for them to feel full so try not to rush them when they are eating
- Follow your child pre-schooler's appetite, some days they may eat a lot and other days not - this is normal. Their appetite changes based on their growth cycles and activity levels.
- Some children test their boundaries by demanding food, not eating food, or being fussy on types of food- it is all normal and part of their social, intellectual and emotional development. If you are concerned that your child is pre-schooler eating too much or too little think about it and record what they have eaten over a week. If you are still worried talk to your GP, CFHN or a Registered Dietitian
- If your pre-schooler goes to Day Care and they enjoy a lunch time meal, they may not be very hungry when they get home- just offer a lighter meal like a boiled/scrambled eggs, omelette, sandwich, steamed vegetables or salad
- Offer meals your family eat- especially food eaten in your culture. It is also good to try foods from different cultures like curries or stir-fry

Do you have any concerns about your pre-schooler's appetite or eating?

- Yes
- No

You might experience your pre-schooler's appetite to be different every day. Some days they will only eat a few bites and other times, they will eat more than they normally do. It is very important that you do not try to force your pre-schooler to eat. This can make them lose the skill to know when they are hungry or full and as they grow older it may lead them to eat more than what their bodies need. Instead, we need to let them lead and take control. You can try the following:

If you can, try to eat at the same time each day - pre-schoolers love routine and they will learn what to expect. Regular meal times ensures your pre-schooler has the opportunity to eat before they become too hungry or too tired - which can both lead to rejection of foods.

Offer 3 meals and snacks every 2 to 3 hours in between - it is helpful if you eat with your pre-schooler and not only focus on feeding them. Let them feed themselves - this is an important skill that they need to learn - it will be messy, but with practice they will get better. The mess is important for their learning - to eat food and to touch, feel and explore what they are eating. This makes for a healthy relationship with food.

- - - try to eat at set times instead of eating small things all day as they won't get hungry to eat their main meals
    - let your pre-schooler take the lead by offering foods you choose during meal and snack times – You provide, they decide
    - at meals and snack times let your pre-schooler decide how much and whether they eat from foods you have offered
    - Make sure your child is not drinking too much milk – this fills them up and stops them being hungry for foods
    - Let your pre-schooler feed themselves in their own way - fingers or utensils, fast or slow, a lot or little, 1 or 2 foods

if your pre-schooler doesn't eat the meal or snack you have prepared, stay calm and relaxed, pack away and offer the next food at the next meal or snack time.

If your pre-schooler does not like some foods, try to re-offer that food again on another day instead of limiting the menu to only foods they like. This encourages them to continue to only eat certain foods and enables food fussiness.

If your child doesn’t eat part of the meal – for example, the vegetables, this is their choice. You don’t need to offer extra serves of other food – for example, meat – to make up for not eating their vegetables pre-schooler

Avoid bribing or punishing for not eating certain foods. Try to avoid using food as a treat or reward for polite/good behaviour – use positive verbal praise and hugs instead

Try new foods or meals and have family or friends over for meals

Talk to them about food preparation and let them help you in the kitchen e.g playing with vegie scraps, pretending to cook, play with plastic bowls / utensils, set the table, etc.

When you notice that they are eating well at the table, tell them what they are doing well and how pleased you are

e.g. "I like how you have tried the new curry..."

Accept their choice if they are not hungry - avoid following them around and placing food in their mouth.

Is your preschoolerpre-schooler still

Do you offer drinks other than water or cow's milk?

(soft drink / juice / flavoured milk)

- Yes
- No

If so, what do you offer?

Do you need some information around drinks? Do you need some information around drinks?

- Yes
- No

Advice on Appropriate Drinks

Remember that cow's milk should be low fat now that your child is over 2 years.

Your child should not be given any fruit juice or any soft drinks, no energy drinks, no flavoured milks or flavoured waters. Children should not be offered tea or coffee due to the caffeine/tannin content.

Water is the best drink in their cup.

Are you cleaning your child's teeth twice a day, i.e. after breakfast and before bed, with a low fluoride toothpaste? It is important that you are cleaning your child's teeth up until the age of 8 years - as they cannot do this successfully by themselves.

- Yes
- No

Dentists recommend that parents help clean their child’ s teeth until the age of 8 years old, as children are developmentally unable to clean well enough by themselves. You can let your child have their turn and then check afterwards, so your child is learning how to clean their teeth properly. Children’s teeth should also be flossed. Talk to your dentist if you are unsure how to do this.

Water is the best drink to keep teeth healthy.

They also need a yearly dental check-up.

Has your child had their annual dental check-up? Yes/No

Teething and Dental Advice

It's a good idea to check your preschooler's teeth by lifting his/her lip and checking that the teeth look white and healthy. To keep the teeth healthy use a baby toothbrush and toothpaste (low fluoride) twice a day to remove the plaque which causes tooth decay.

A dental examination for your pre-schooler is recommended from 1 year of age. After that, you need to talk with your dentist about how often your child needs a check-up because everyone has different oral health needs and risk levels which determine how often they should have a check-up. This helps your pre-schooler get used to having their teeth checked and ensures their teeth and gums are healthy.

For dental health options go to: [www.health.gov.au/dental](http://www.health.gov.au/dental) Or see your local / family dentist

Comments

Is there anything you would like to ask me before we set a goal and move on the next section?

Do you find grocery shopping with your pre-schooler challenging? Yes No

- Prepare a shopping list – and stick to it! Try not to get distracted by the unhealthy foods in shop and buy anything ‘extra’
- Set expectations with your child before your trip. For example, rewards them with a trip to playground if they do not ask for unhealthy snacks. Take healthy snacks and water bottles to drink
- Eat a healthy snack before shopping so you and your child are not hungry and ends up buying unhealthy snacks
- Fill your trolley with mostly seasonal fresh produce like fruit and vegetables
- Try to avoid the aisle with lollies, soft drinks, biscuits & chips
- Buy frozen or canned fruit and vegetables to save time and money.
- How often does you child pass a bowel motion? Or - does you child pass a regular bowel motion easily?

_______________________________________

Some children go to the toilet to pass a stool (poo, faeces) 2 or 3 times a day, some poo 2 or 3 times a week; all of these can be normal as long as the poo is soft and easy to pass.

Constipation is when your child’s poo is hard, painful or difficult to pass. If your child becomes constipated they may feel pain and discomfort and may avoid pooing, which can make the problem worse. Some children who are really constipated can also have runny, liquidy poo that they can’t control (overflow) which passes around the hard, old pieces of poo in the bowel. This can cause soiling of underpants. Hard poo that is difficult to pass can also cause overstretching of the anus, which can lead to tearing of the skin around the anus.

To prevent constipation:

- - Ensure enough fibre in your child’s diet. Foods that are high in fibre include wholegrain breads and cereals – oats, brown rice, brown pasta, legumes, beans, fruit and vegetables. Whole fruit and vegetables (not juice) contain more fibre
  - Encourage lots of water to drink
  - Reduce cows milk to no more than 500 mls a day
  - Be physically active everyday
  - Read children’s books with your child about going to the toilet
  - Encourage your child to sit on the toilet at least once a day – about 20 minutes after meal times is a good time as this is when the body feels the natural urge to pass a poo. Your child may need a footstool to encourage good positioning on the toilet, which makes it easier to pass a poo.
  - Teach your child to go to the toilet when they feel the urge/need to go (and not to hold on!).
  - Use positive words, remain calm if they have an accident, start a sticker or reward chart to encourage their attempts and praise your child for sitting on the toilet and trying
  - It is very important that a child is encouraged in correct toileting behaviour and not criticised or punished.

If your child’s poo is already hard despite the measures above, or you are concerned your child is constipated, the skin around their anus is damaged, they are ‘holding on’ or have frequent soiling of their underwear see your GP for help. Your child may require some medication or further assessment – but your GP will advise you regarding this.

- For more information see :

<https://raisingchildren.net.au/babies/health-daily-care/poos-wees-nappies/constipation>

- [www.continence.org.au/who-it-affects/children/soiling](http://www.continence.org.au/who-it-affects/children/soiling)

**SET NEW GOAL FOR PRE-SCHOOLER'S FOOD & FEEDING**

T11 goal for [demographics_arm_1][baby_name]'s food & feeding=[stage_11_arm_1][new_fgoal_t11]

T12 goal for [demographics_arm_1][baby_name]'s food & feeding=[stage_12_arm_1][cnutri_goal_t12]

Let's set a new goal together for your feeding of [demographics_arm_1][baby_name].

(e.g., Aim to eat together as a family at least once a day /

Aim to avoid junk food / Aim to have healthy snacks cut

up and ready to go / minimise takeaway foods / avoid soft drinks, juices and flavoured milks / offer

healthy snacks in between meals / sit at the table with the family for meals whenever possible / Use cups for all drinks. / Offer 1 serve fruit and 2.5

serves veg each day. / Try to decrease the amount of fast foods / junk foods offered./ Aim to not use

food as a reward. Aim to have water as their main drink. )

Avoid junk food aisles in the supermarket / Praise your child for good behaviour while shopping

(THIS INFO WILL BE EMAILED TO

[demographics_arm_1][mothers_first_name].

PLEASE WRITE FULL SENTENCES WITH NO ABBREVIATIONS)

**KEY AREA 2- YOUR PRE-SCHOOLER & PHYSICAL ACTIVITY**

T11 goal for [demographics_arm_1][baby_name]'s physical activity=[stage_11_arm_1][cpa_goal_t11]

T12 goal for [demographics_arm_1][baby_name]'s physical activity=[stage_12_arm_1][cpa_goal_t12]

The aim of this module is to provide information on how parents can keep their pre-schooler active as they transition into being more active and independent.

Key Messages

"Keep me active and I don't need screen time for play"

We are going to talk about [demographics_arm_1][baby_name]'s physical activity now.

Last time we spoke we discussed Fundamental Movement Skills,

In the booklet there is new information about fundamental movement skills.

**Screen time**

Pre-schooler’s should spend at least 180 minutes a day in a variety of physical activities. This should include at least 60 minutes of energetic activities such as running, jumping , kicking and throwing spread across each day.

By turning off any screens including TV, DVDs, using computers, iPads, tablets, smart phone or any other electronic devices you are enhancing your child's learning and development.

A child's vocabulary is actually increased by talking with you and family members - too much use of any screen can lead to speech issues for your child and a lower vocabulary overall (use of words).

At this age it is recommended to have no more than 1 hour of screen time a day.

How much screen time would your pre-schooler have each day

- Less than 1 hour (hours )?
- More than 1 hour
- More than 2 hours More than 3 hours

Screen time

Some parents might place a screen in front of their child to give them time to do house chores or to keep their children settled and quiet. There are however so many other things that you can use to keep your child entertained with active or quiet play.

Do you need some ideas for alternatives to screen time?

- Yes
- No

**YOUR PRE-SCHOOLER’S PLAY- 3 hours a day**

Pre-schoolers should spend at least 180 minutes (3 hours) a day of physical activities. This should include at least 60 minutes (1 hour) of energetic play such as running, riding, climbing on play equipment, kicking and throwing, spread throughout the day.

Less energetic activities like putting toys away, helping with everyday household tasks, and walking, all count for movement too.

Keep doing or introduce activity to your daily routine to develop healthy habits for life. For example, go for a quick walk after breakfast around the block or have a dance to a few fun songs at home.

Outdoor play is important for your child’s physical and emotional development and wellbeing. They have more freedom to make noise, a mess and move around on different surfaces. Playing in the outdoors helps to promote a calming and happy mood, build self-esteem and confidence, and develop balance, coordination and strength. Energetic play outside can help them sleep better too.

**SET NEW GOAL FOR ACTIVITY/SCREEN TIME**

T11 goal for [demographics_arm_1][baby_name]'s activity and screen time= [stage_11_arm_1][cpa_goal_t11]

T12 goal for [demographics_arm_1][baby_name]'s activity and screen time= [stage_12_arm_1][cpa_goal_t12]

"Let's set a goal together for [demographics_arm_1][baby_name] 's activity including screen time."

E.g. aims to practice balancing, jumping and kicking with [demographics_arm_1][baby_name].

Aims to minimise screen time for pre-schooler to less than 1 hour.

Aims to have 3 hours of supervised active play per day.

Try some quiet play or craft activities instead of screen time.

Keep practising fundamental movement skills most days.

Join a playgroup for some active play time

Go to the park a few days a week for active play. Aim to involve my child in growing some vegetables and fruit

Look at raising children

-

https://raisingchildren.net.au/pre-schoolers/play-learning/screen-time-media/healthy-screen-time-2-5-years Resources <https://karitane.com.au/parents-resources>

Our playtime:

[https://www.youtube.com/playlist?list=PLbw1KgO1-UrEIIA-Tku4JjRx5E_WvSBNP](http://www.youtube.com/playlist?list=PLbw1KgO1-UrEIIA-Tku4JjRx5E_WvSBNP)

Bilingualism

https://raisingchildren.net.au/pre-schoolers/connecting-communicating/bilingualism-multilingualism https://raisingchildren.net.au/preschoolers/play-learning/play-preschooler-development/emotions-play-preschoolers

(THIS INFO WILL BE EMAILED TO

[demographics_arm_1][mothers_first_name].

PLEASE WRITE FULL SENTENCES WITH NO ABBREVIATIONS)

Do you have any other questions before we move on to the next topic?

**KEY AREA 3- YOUR PRE-SCHOOLER’S SLEEP**

Key Messages

"I'm a pre-schooler now not a baby, I can sleep at night without any milk"

Are you having any issues with your pre-schooler’s sleep behaviour?

- Yes
- No

Does your pre-schooler have a regular bedtime routine and bedtime?

- Yes
- No

If no, would you like a suggestion on a pre-schooler bedtime routine?

- Yes
- No

SLEEP

Bedtime routine

pre-schoolerHaving a bedtime routine can help you both get the right number of hours of sleep and to have energy to do your day to day activities.

Bedtime routines are important for 3 – 4 year olds. Introducing "quiet time" before bed can help your child relax and wind down. Pack away toys and prepare the bedroom as a place of rest. A routine might include a bath, teeth cleaning, story time/ song , cuddle and bed. Say goodnight and tell them it is time to go to sleep.

It is important that your pre-schooler has a regular sleep routine. This means going to bed at the same time each night and waking at same time each morning.

Most pre-schoolers are ready for bed by 7pm. If your child has a much later bedtime it may be hard for them to get enough sleep and this will make them tired and frustrated during the day. That makes your job as a parent more difficult.

To help your child fall asleep earlier:

Aim for no screen time 1 hour before bedtime.

Before putting them into bed, help them wind down by telling them a bedtime story or reading a book

Keep them away from any screens at least 30 – 60 minutes before bedtime such as, TV, computers, phones or tablets close to bedtime. When their eyes are exposed to bright light it can make it harder for your child to settle

Create a calming bedtime routine for them such as having a warm bath, brushing their teeth, sitting in bed and reading them a book

Make sure their room is quiet and if you can lower the bright light or use a lamp or night light

- Before leaving the room, check that your child has everything they need for sleep, like their favourite cuddly toy or blanket and that they’ve been to the bathroom and had a final drink of water.. Remind them to stay quietly in bed
- If your child gets out of bed, calmly ask them to go back to bed. Say that you’re just in the other room. Repeat this firmly but in a calm tone over and over until they don’t get up again Sometimes your child might actually need something. If your child is scared of a monster under the bed, you can quickly check together that there are no monsters under the bed. If your child is scared every night of monsters, you might check the room with your child before they settle into bed as part of the bedtime routine. Children’s books are also a great idea to read together dealing with the issue of monsters. If your child is scared of the dark, think about using a night-light.
- children should sleep in a room that is quiet and dark and without a TV or other screens
- keep bedtime and awake time the same during the week and on weekends Children at this age need 11 – 13 hours of sleep in 24 hours

".

If you are still concerned you may want to talk to your Child and Family Health Nurse or GP. See local C&FHN if needing more support.

* Tresillian (02) 9787 0855 or 1800 637 357

* Karitane (02) 9794 2300 or 1300 227 464

- Raising Children website [www.raisingchidlren.nsw.gov.au](http://www.raisingchidlren.nsw.gov.au)

Setting goal for sleep to be emailed to mother

Sleep goal : examples -

Aim to have a regular bedtime and routine before bed Aim for no screens an hour before bedtime.

Aim to change to a 7pm bedtime

Aim to have some quiet time in the afternoon if pre-schooler no longer having a day time nap

We have now finished talking about [demographics_arm_1][baby_name].

Do you have any questions you would like ask, before we move on to your health and wellbeing?

**KEY AREA 4- YOUR NUTRITION & PHYSICAL ACTIVITY**

The aim of this module is to address barriers in achieving healthy diet.

How are you going with your healthy eating?

T11 goal for your nutrition=[stage_11_arm_1][mnutri_goal_t11]

T12 goal for your nutrition=[stage_12_arm_1][mnutri_goal_t12]

**We are now going to talk about healthy eating**

Eating well is vital to physical and mental well-being, which will affect the way you get through each day, and the way you interact with your family. It is also important that your children see you looking after yourself in this way, as they learn these habits for life - now.


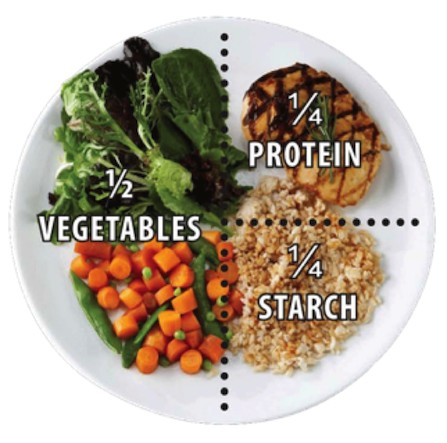


Do you think you are getting enough iron-rich foods in your diet every day?

- Yes
- No

IRON

INFORMATION TO SUPPORT IRON INTAKE

Iron is a vital mineral for the human body and is involved in many bodily functions. Iron assists in producing energy, immune function and the production of haemoglobin. Haemoglobin enables the delivery of oxygen from the lungs to the tissues. Women in reproductive years and pregnant women are at risk of iron deficiency.

Symptoms of iron deficiency include: fatigue, lack of concentration, headache, pale skin, weakness/ dizziness and increased risk of infection. If you are concerned about your iron levels, attend the GP for a consultation and blood test.

Pregnancy is a period of higher nutritional demands, particularly for iron to allow for increase the maternal red cell mass. More iron is required during pregnancy to support the rapid growth of the foetus and placenta. Insufficient iron can increases the risk of premature births or low birth weights for the baby, contributing to undesirable short and long-term health impacts.


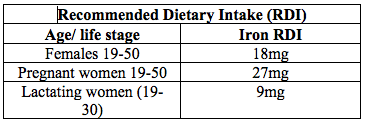


Iron absorption can be enhanced by eating foods that are high in vitamin C at the same time.

Foods high in Vitamin C include tomatoes, capsicum, broccoli, oranges, and berries. Cooking plant foods can also increase the amount of available iron. Avoid tea, coffee or calcium rich foods directly after consuming foods containing iron. Ask your GP if any of your medications or herbal supplements can impair iron absorption.

Iron Supplementation

Iron is available in many dietary supplements such as multivitamins with iron. High dose of supplemental iron (45mg/day or more) may lead to gastrointestinal side effects such as nausea and constipation. Natural forms of supplements may have fewer gastrointestinal side effects however usually offer a smaller dose of iron.

There are two types of iron:

Haem iron is derived from animal products such as lean meats and seafood. Haem iron is easily absorbed by the body. Non-haem iron is in plant-based foods such as nuts, beans, vegetables, and fortified grain products. It is not absorbed as easily as haem iron to our bodies.


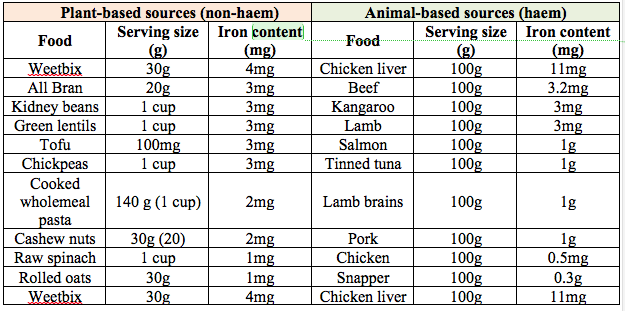


Food tips to increase iron dietary intake

• Breakfast ideas

o Have 2 pieces of iron fortified Weetbix

o Rolled oats with berries

o Wholegrain toast with peanut butter and a piece of fruit

o Wholegrain toast with eggs and tomato

• Lunch ideas - try having a vitamin C rich piece of fruit with lunch too!

o Wholegrain sandwich or wrap with tuna and salad

o Four-bean mix salad with wholegrain roll or crackers

o Roasted vegetable, fresh tomato , nut and baby spinach salad

• Dinner ideas

o Beef/lamb/kangaroo/pork steak with steamed vegetables

o Stir-fry or curry containing chicken/pork/tofu/cashews/pine nuts and vegetables served with brown rice

o Mince with grated vegetables, pasta and tomato based sauce

• Snack ideas

o Wholegrain crackers with hummus and tomato

o Small can of baked beans

o Roasted chickpeas (olive oil, paprika and cumin)

[https://www.eatforhealth.gov.au/food-essentials/how-much-do-we-need-each-day/sample-meal-plan-women](http://www.eatforhealth.gov.au/food-essentials/how-much-do-we-need-each-day/sample-meal-plan-women)

Do you think you are getting enough calcium in your diet every day?

- Yes
- No

CALCIUM

INFORMATION TO SUPPORT WITH CALCIUM INTAKE

Calcium is a one of the most abundant and vital minerals in the human body. Adequate calcium is important during all stages of life. It is required for growth and maintenance of the skeleton, including bones and teeth. The range of other functions includes muscle contraction, heart function, bone formation, hormonal regulation, nerve and enzyme functioning.

Risks of deficiency

A low intake of calcium is associated with an increased risk of osteoporosis, which means low bone density. Fragile bones are at a greater risk for fractures from bumps or falls. If limited calcium is consumed, the body will use calcium stores from bones, resulting in weak and brittle bones.

Nutrient and food compound interactions with calcium absorption

Foods with high oxalic acid and phytic acid include green leafy vegetables, beans, nuts and wholegrain products. These foods can bind to calcium and inhibit the absorption if consumed at the same time. For people eating a variety of healthy foods, these interactions are likely to have little nutritional impact overall.

Vitamin D

Vitamin D works with calcium to transport and aid the absorption of the mineral. Vitamin D can be sourced from sunlight, foods and supplements. Fish such as tuna and salmon contain Vitamin D and fortified food products.

Vitamin D deficiency affects over one in five Australian adults. Having adequate calcium and Vitamin D levels can reduce the risks of fractures.

Physical activity for Healthy Bones

Bones provide a strong structure for our body, they support our muscles which allow us to move, protect our organs and store calcium. Bone development continues throughout adulthood, so it is important to maintain bone health as we grow older. Bones respond according to changing lifestyles and including regular physical activity into our routines will promote bone cell growth, leading to stronger, healthier bones.

A few exercise tips to maintain bone strength:

Try doing 10 vertical jumps before breakfast, lunch and dinner

- 10-30 minutes of continuous brisk walking or jogging - you could mix it up by alternating
- 2-minutes of walking with 30-seconds of jogging
- 2 sets of 10 repetitions of "sit-to-stands" or squats - challenge yourself by holding 500g food cans in each hand or increasing the sets!
- Playing with your children is great for your bones - jumping/bouncing games such as skipping or hopscotch are fun options

Pregnancy

Adequate intakes and stores of calcium during pregnancy can decrease adverse gestational outcomes, such as hypertensive disorders like pre-eclampsia. Pre-eclampsia is associated with a significant number of maternal deaths and risks of preterm birth.

An inadequate dietary intake of calcium increases the risk of depleting maternal skeletal stores and consequently adversely effecting calcium equilibrium. In the third trimester a large amount of calcium is transferred to meet the demands of the growing foetal skeleton. During breastfeeding and lactation there is a loss of calcium in the breastmilk, therefore the dietary intake requirements are increased.


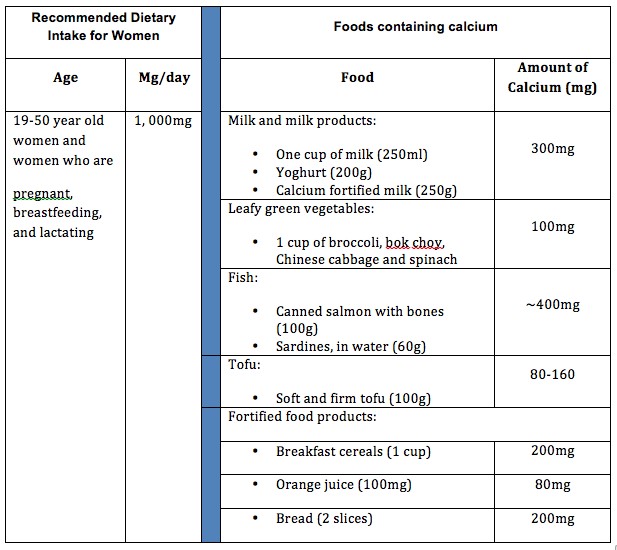


Food tips to increase calcium dietary intake

- - Have a glass of milk with breakfast and calcium fortified toast
  - Start the day with milk and calcium-fortified breakfast cereals
  - Add muesli and fruit to yoghurt
  - Swap sour cream for low-fat Greek yoghurt
  - Use milk and yoghurt to make a fruit smoothie
  - Try using firm tofu in a stir-fry or curry and add nuts for crunch
  - Scramble soft/ silken tofu instead of scrambled eggs
  - Snack on cheddar or other hard cheeses with vegetable sticks or crackers
  - Have a handful of nuts as a snack
  - Have a tuna or salmon salad for lunch
  - Try tuna or salmon pasta and vegetable bakes

For more information on

Healthy eating guidelines : [https://www.eatforhealth.gov.au/guidelines/australian-guide-healthy-eating](http://www.eatforhealth.gov.au/guidelines/australian-guide-healthy-eating) Have a look at the www.eatforhealth website

Referral to Get Healthy made?

- Yes
- No

Get Healthy Referral - 1300 806 258 [https://www.gethealthynsw.com.au/](http://www.gethealthynsw.com.au/)

**SET NEW GOAL FOR MUM'S NUTRITION**

T11 goal for your nutrition=[stage_11_arm_1][mnutri_goal_t11]

T12 goal for your nutrition=[stage_12_arm_1][mnutri_goal_t12]

"Let's set a new goal together for your nutrition."

(THIS INFO WILL BE EMAILED TO

[demographics_arm_1][mothers_first_name].

PLEASE WRITE FULL SENTENCES WITH NO ABBREVIATIONS)

Aim to increase my daily iron and/or calcium intake. Make water my main drink.

Decrease unhealthy snacking.

Aim to increase my daily fruit / vegetable intake

Have a look at the www.eatforhealth website

The aim of this module is to address barriers in achieving healthy physical activity levels.

Are you managing any physical exercise at the moment?

It can sometimes be harder to fit exercise into the day when you are busy with a pre-schooler. Even a little bit of activity is better than doing none at all and it will boost your energy and wellbeing.

Some ideas for fitting exercise into your day include:

•Doing a YouTube video or exercise DVD at home - a 5-10minute one is good to start with - you can try it when your pre-schooler is sleeping - or even do it when your pre-schooler is in the room playing.

•Join an online virtual exercise trainer

•Going for a walk with your child / play with them at the park / kick a ball / play chasing games / run with them while they ride their scooter or bike.

•Dance around the house with your pre-schooler

•Walk whilst you are on the phone - don't sit and talk!

•If you are at work try taking the stairs instead of the lift and going for a walk at lunchtime. Get off the bus one stop earlier or park further away so you get to walk some more.

•If you have another adult living in your house - try going for a 10minute jog in the morning before your pre-schooler gets up

Try developing a healthy routine by finding different physical activities that your pre-schooler and the family can spend time doing. For example:

•Physical activity: develop an exercise routine with the family even it is only a 10-15 minute walk every day

•Team sport: many places offer team sports for children as young as pre-schoolers and for adults at any age like swimming, tennis, soccer or gymnastics. It is beneficial for both and a good way to have scheduled physical activity in your week. It can also give you motivation and confidence to keep fit and to meet new people

For more ideas try: https://www.makehealthynormal.nsw.gov.au/ https://www.gethealthynsw.com.au/

**SET NEW GOAL FOR YOUR PHYSICAL ACTIVITY**

T11 goal for your physical activity=[stage_11_arm_1][ mpa_goal_t11]

T12 goal for your physical activity=[stage_12_arm_1][mpa_goal_t12]

"Let's set a new goal together for your physical activity."

E.g. will aim to start doing some exercise?

Aim to do some exercise at home most days? / Will aim to be active most days for around 30 minutes / will

try doing exercise video's at home/ Try to run with my pre-schooler 3 times a week at the local park/ Aim to join in a team sport or exercise class)

(THIS INFO WILL BE EMAILED TO

[demographics_arm_1][mothers_first_name].

PLEASE WRITE FULL SENTENCES WITH NO ABBREVIATIONS)

**KEY AREA 5: MENTAL WELLBEING/SOCIAL SUPPORT**

T11 goal for mental wellbeing/social support= [stage_11_arm_1][ mmental_goal_t11]

T12 goal for mental wellbeing/social support= [stage_12_arm_1][mmental_goal_t12]

Note

The aim of this module is to provide support to mother around their emotional wellbeing. To offer resources to gain more support if needed.

"Now we will be discussing your emotional health and self care."

We ask about your emotional well-being because, as a parent, it can be common to experience a range of emotions, or changes in mood - which may include feeling overwhelmed/ low mood / anxiety / frustration /anger or sadness to happiness and joy.

The previous Healthy Beginnings booklets from birth to 4 3 years have tips on how to look after your overall wellbeing. You might know what is good for you but it is important that you remember to always have some time to practise it.

Each day set aside some time, even if it is just 5 minutes but preferably longerfor yourself. Choose something you enjoy that brings a smile to your face. By caring for yourself, it will help you to keep going with your day to day activities whether it is work, looking after your child or the family, or to do house chores. Caring for yourself is also really good role modeling for your children to see for their future mental health.

Activities for your own self-care do not have to be costly. It might be as simple as sitting in the sunshine, talking to a friend, going for a jog, YouTube a yoga session, reading a book, listening to your favourite music or painting your nails.

Parenting can be exhausting and challenging so if you ever feel like you need support or help reach out to your friends or family. If you are not enjoying your usual activities, feeling overwhelmed or experiencing a persistent low mood, talk to your Child and Family Health Nurse, GP or one of the services below.

How has your mood been over the last 2 weeks?

Is this something new for you?

Yes

No

Do you need any support with this? Do you need any support with this?

Yes

No

No

Help services - contact details

Help and support is available if you are feeling overwhelmed, stressed or more anxious than usual. Getting support will help to stop the worries, help with perspective on your thoughts and challenge any unhelpful thinking styles that may be emerging.

Supports include: BeyondBlue - 1300 224 636

Healthdirect - 1800 022 222

Lifeline on 131 114

Local Child and Family Health Centre

GP - for Mental Health Care Plan and counselling referral

Tresillian or Karitane pre-schooler

Support Line- 1300 224 636 (info and advice from a trained Mental Health Professional)

Helpline - 1800187 263 (9am - 5pm weekdays - Mental Health info and referral support services). SANE Australia: [www.sane.org](http://www.sane.org/)

Parentline: 1300 130 052 - telephone counselling with kids aged 0 - 18 yrs

NSW Family and Community Services : [www.community.nsw.gov.au/parents-carers-and-families](http://www.community.nsw.gov.au/parents-carers-and-families)

Family Relationships Advice Line : 1800 050 321 - 8am - 8pm, Mon. - Fri. and 10am - 4 pm Sat. Family Relationships Online: [www.familyrelationships.gov.au](http://www.familyrelationships.gov.au/)

Family Referral Service: [www.familyreferralservice.com.au](http://www.familyreferralservice.com.au/) South East and Northern Sydney: 1800 066 757

South West Sydney: 1300 244 826

Western Sydney: 1300 403 373

Mensline Australia: 1300 789 978

Relationship Australia: [www.relationships.org.au](http://www.relationships.org.au/)

Resourcing Parents: <http://www.resourcingparents.nsw.gov.au/> Karitane care line: 1300 227 464

Tresillian: 1300272 736

Parent works: [www.parentworks.org.au](http://www.parentworks.org.au/) - online modules through Sydney Uni., for parent with kids aged 2 -16 yrs D.V. Line: 1800 656 463

Child Protection Helpline: 13 21 11

Link2home Homelessness: 1800 152 152

Black Dog Institute: [www.blackdoginstitute.org.au](http://www.blackdoginstitute.org.au/)

MindSpot: 1800 61 44 34 - Online assessment and treatment for anxiety and depression Head to Health: [www.headtohealth.gov.au](http://www.headtohealth.gov.au/) - digital mental health resources

QLife: 1800 184 527: LGBTI services

Mens Line Australia: 1300 789 978

Carers Australia: 1800 242 636 [www.carersaustralia.com.au](http://www.carersaustralia.com.au/)

Make healthy normal website: [https://www.makehealthynormal.nsw.gov.au/](http://www.makehealthynormal.nsw.gov.au/)

Emotional health and self care

Looking After Yourself

Why is it so important to look after yourself?

The job of parenting may be one of the hardest things you will ever have to do. Parenting often involves being on call 24 hours a day, with no sick leave, no days off and requiring endless amounts of love and patience. Parenting doesn't come with a manual; most parents are learning on the job.

When was the last time you had a health check-up?

- Yes
- No

What self-care can look like - it does not have to be expensive or for long periods of time - self-care can be free of charge and can be for 10 - 30 minutes a few times a week - or even weekly. It depends on you and what your needs are. Find those 10 minutes - as it can make a huge difference to your emotional health and well-being. You deserve it. Part of this is actually giving yourself permission to do it


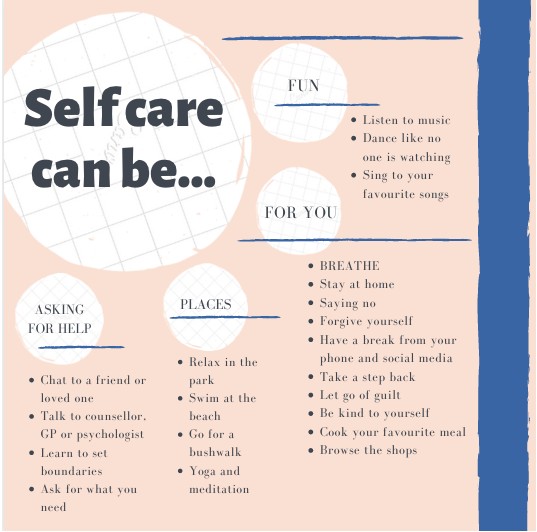


If you are experiencing a persistent low mood, feel overwhelmed, or the stress in your life is interfering with your usual activities, please speak to your child and family health nurse or GP or seek support through one of the services listed below.

Resources to support your parenting and you!

[www.beyondblue.org.au](http://www.beyondblue.org.au/) beyond blue provides information and support to help everyone in Australia achieve their best possible mental health, whatever their age and wherever they live.

[www.resourcingparents.org.au](http://www.resourcingparents.org.au/) government website with information about free parenting courses in your area (NSW)

[www.raisingchildren.net.au](http://www.raisingchildren.net.au/) government website with evidence based information about all aspects of childcare and parenting

[https://www.facs.nsw.gov.au/families/parenting](http://www.facs.nsw.gov.au/families/parenting) NSW government website with practical tips on parenting and raising children

1800RESPECT - 1800 737 732

The national family violence and sexual assault counselling service is available 24 hours a day, 7 days a week. It's confidential and free to call.

https://mnclhd.health.nsw.gov.au/media-releases/mental-health-access-line-1800-011-511/

Anyone with a mental health concern can call this number 24 hours a day to talk to a mental health professional for support.

[www.tresillian.org.au](http://www.tresillian.org.au/) Tresillian is Australia's largest child and family health organisation providing expert parenting advice to families during the early years.

[www.lifeline.org.au](http://www.lifeline.org.au/) Lifeline is a national charity providing all Australians experiencing a personal crisis with access to 24 hour crisis support and suicide prevention services. 13 11 14

**LET'S SET GOAL FOR MUM'S Emotional WELLBEING**

T11 goal for your emotional wellbeing =[stage_11_arm_1][ mmental_goal_t11]

T12 goal for your emotional wellbeing=[stage_12_arm_1][mmental_goal_t12]

(THIS INFO WILL BE EMAILED TO

[demographics_arm_1][mothers_first_name].

PLEASE WRITE FULL SENTENCES WITH NO ABBREVIATIONS)

Take time out for self-care /Aim to plan some self-care / Aim to find something that helps me to relax / Take time out for myself / Try a mindfulness exercise/ Smiling minds app or the Head space apps for relaxation. / Find a strategy for dealing with

my frustration with my pre-schooler. Get support and information about pre-schooler behaviour and relationships.

Additional Supportive Information:

Additional Supportive Information to be emailed for mother to support parenting e.g. play group contact, CFHN contact, e.g. Circle of Security, Triple P, Quitline, Family Referral Service, Resourcing Parents.

(Please write 'No' if there is no additional supportive info)

Any referrals made or information given or extra goals sent to Mum:

(To be sent to Mum with the goals)

Comment:

**CLOSING THE PHONE CALL**

"[demographics_arm_1][mothers_first_name], it's been great to talk with you and discuss how you're feeling about your and [demographics_arm_1][baby_name]'s health."

Now recap goals below:

Pre-schooler's food and feeding goal=[stage_13_arm_1][cnutri_goal_t13]

Pre-schooler's PA & Screen time goal=[stage_13_arm_1][cpa_goal_t13]

Pre-schooler's sleep goal=[stage_13_arm_1][csleep_goal_t13]

Mum's nutrition goal=[stage_13_arm_1][mnutri_goal_t13]

Mum's PA goal=[stage_13_arm_1][mpa_goal_t13]

Mum's emotional wellbeing goal=[stage_13_arm_1][mmental_goal_t13]

Additional supportive info to mother=[stage_13_arm_1][more_info_t13]

Key Messages

- - Keep me healthy, give me fruit and vegetables every day
  - Water in a cup is best for me and my teeth
  - Keep me active, I don't need screen time for play
  - I'm a pre-schooler now, not a baby, I can sleep at night without any milk
  - Reward me with cuddles and praise, food rewards are unhelpful for me

"Thank you [demographics_arm_1][mothers_first_name] for your time today. You will hear from Healthy Beginnings again in a few months. .

In a moment you will receive an email which summarises our discussion and the goals we set together. You can refer to these anytime.

Any additional comments or notes

Time finished

Total Time
