# Supplementary material for: Effectiveness of a telephone-based randomised clinical trial targeting obesity risk of preschool-aged children: An extension study during the COVID-19 pandemic
Source: Int J Obes (Lond). 2025 Aug 14;49(11):2241–8. doi: 10.1038/s41366-025-01869-4 (PMC12583190; doi:10.1038/s41366-025-01869-4)
Supplement: Supplementary file 6 — Supplementary Document 3_3-4 years_SMS [file 41366_2025_1869_MOESM6_ESM.docx]

| **SMS Scheduling CHAT Study**  **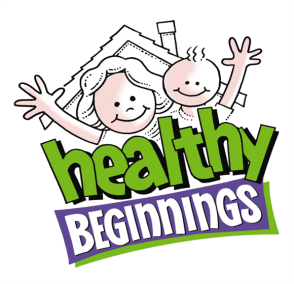**  **32 36 MONTHS SCHEDULE** | | |
| --- | --- | --- |
| **Reference date:**  **Date of birth** | **DAYS after ref date** | **Message content** |
|  | 1042 | Hi [FirstName]! We hope you are finding the Healthy Beginnings program helpful. In the next couple of months you will receive text messages that will provide health tips to support you and [BabyName].  Our nurses will give you a call soon. Send us a text if you have any questions, and we will get back to you. |
|  | 1045 | With busy lives it can be really hard to eat a meal together but it is so important. Family meal times can help [BabyName] by: seeing you as a role model eating healthy food, encouraging them to eat family foods and to accept different foods and also teaches them to be social by learning to talk and have a conversation. |
|  | 1049 | As an adult we need to get 5 serves of vegetables in each day.   - boil carrots, peas, corn or broccoli and have them as snacks - cut up carrot, celery or cucumber sticks and dip them in hommos - Throw extra vegetables in your pasta/curries/salad   1 serve = ½ cup of carrots, 1 medium tomato, ½ cup cooked broccoli or spinach, 1 cup green leafy vegetables, 1/2 medium potato |
|  | 1052 | Try and do something different this weekend with [BabyName] and the family:   - Pack their scooter/ bike, have a BBQ/ picnic and   - go to the bike park   - go the local park   - walk along the beach, or   - go for a bush walk |
|  | 1056 | Don’t forget to look after yourself [FirstName]. If you are stressed it can be hard to think properly and respond in a calm and supportive manner to your toddler. It can help if you eat healthy food, keep up your physical activity, find something that helps you to relax and take time out for yourself and for your relationship with your partner or other close friends. |
|  | 1059 | The Healthy Beginnings booklet has information on Fundamental Movement Skills. This is really important for [BabyName]’s development. To help you with the steps and teach [BabyName] to catch, check out this video <https://www.youtube.com/watch?v=sO7ZAzlhdxk&feature=youtu.be> |
|  | 1063 | Hi [FirstName], do you have any questions about eating, physical activity or sleeping? Our nurses can help.  If you haven’t spoken to a nurse yet and you would like to, text back YES and we will contact you. |
|  | 1066 | Bottle feeding can lead to tooth decay and ear infections in toddlers. If you need support so [BabyName] can stop bottle feeding and start using a cup, text YES, and a nurse will be in touch. |
|  | 1070 | Need some personalised support with healthy eating and physical activity? Join Get Healthy, a free service that can support you. If you would like to join text YES or visit <https://www.gethealthynsw.com.au/> or Call 1300 806 258 and chat with a health coach. |
|  | 1073 | Pack a lunch box of healthy snacks for [BabyName] every day. You can boil some eggs, cut up carrot, cucumber, celery and cheese sticks, keep your roasted or steamed left over vegetables, warm them up the next day, boil ½ cup of frozen peas and corn, make fruit skewers. |
|  | 1077 | Looking for new ways to get your physical activity in each day. We need at least 30min of physical activity every day. This can be 30 minutes in one go or broken up into 10 minutes sessions throughout the day. Start with 10 minutes and slowly build this up. See Make Healthy Normal for some ideas <https://www.makehealthynormal.nsw.gov.au/Pages/getting-active.aspx> |
|  | 1080 | ‘Sometimes foods’ like squeeze pouches, take away food, muesli bars, chip packets, biscuits and crackers are high in sugar, salt and unhealthy fats. [BabyName] might start seeing these foods on TV from family, friends or shops and may want them. It can be hard to say no all the time but try to have a rule around sometimes food like having only small serves less than 1 – 2 times in a week and finding fun ways to reward [BabyName] instead of using ‘sometimes’ food. |
|  | 1084 | It’s worth thinking about whether or not you have a balanced lifestyle. You may have lots to do like looking after the family, house chores, working, social life or volunteer work. If you feel tired and stressed, you may need to try changing some things to help you feel relaxed and healthy. This is good for you and your family. Check out the Healthy Beginnings booklets for tips. |
|  | 1087 | Have a relaxing bedtime routine such as reading books. No screen time for 1 hour before bedtime will help your [BabyName] to get ready for sleep. Children should sleep in a room that is quiet and dark and without a TV or other screen. |
|  | 1091 | [FirstName] will need to do a Personal Health Record (blue book) check at 3 years. Make an appointment with your Child and Family Health Nurse or GP. |
|  | 1094 | Congratulations [FirstName], [BabyName] is now a pre-schooler! Thanks for taking part in Healthy Beginnings program over the last 3 years. We hope it has been helpful. We will be in touch again when [BabyName] is around 40 months old. |
|  | 1124 | Hi [FirstName], the Healthy Beginnings team will be in touch again when [BabyName] is 40 months. You will receive a telephone support call from our clinicians and further text messages.  If you have any questions before then, send us a text and we will contact you. |
|  | 1154 | Hi [FirstName], the Healthy Beginnings team will be in touch again when [BabyName] is 40 months. You will receive a telephone support call from our clinicians and further text messages. |
|  | 1184 | Hi [FirstName], the Healthy Beginnings team will be in touch again when [BabyName] is 40 months. You will receive a telephone support call from our clinicians and further text messages. |

| **SMS Scheduling CHAT Study**  **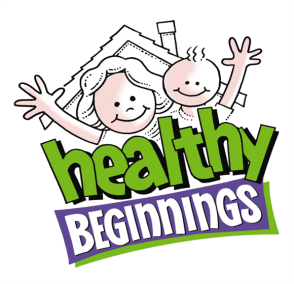**  **41-42.5 MONTHS SCHEDULE** | | |
| --- | --- | --- |
| **Reference date:**  **Date of birth** | **DAYS after ref date** | **Message content** |
|  | 1247 | Hi [FirstName], thank you for your continuing participation with the Healthy Beginnings program. In the next couple of months, you will receive text messages and a telephone support call with tips on yours and [BabyName]’s health and wellbeing. Please text back to book in for your telephone support call. |
|  | 1250 | How often do you sit down for a family meal? Eating together as a family as often as possible is so important for [BabyName]. It helps reduce fussy eating, offers you a chance to be a healthy eating role model, strengthens family bonds, and improves [BabyName]’s language development and communications. |
|  | 1253 | Hi [FirstName], under the current COVID-19 circumstances, it is understandable to feel anxious, exhausted or overwhelmed. To be able to support and also to be a role model for [BabyName], it is important to manage your own emotions. Some useful strategies:   - drink a cup of tea in the sun - sit on the grass and do some deep breathing - talk to supportive friends or family - read a book, do yoga, take a nap, or do something you enjoy |
|  | 1259 | Aim for more movement in your day! The recommendation for [BabyName] is 180 minutes of physical activity every day. Try:   - walking to and from day care/school, or park your car further away to get in more steps - put music on & dance with [BabyName] during breaks (if you are working from home), or after work |
|  | 1262 | Has [BabyName] had their 3 year Developmental (Bluebook) Check with your local Child & Family Health Nurse or GP? If not, now is the time.  Also, don’t forget about [BabyName]’s yearly dental check-up with either your local family dentist or Community Dental Health Clinic. Consider including dental visits as part of your regular health checks. |
|  | 1265 | Hi [FirstName], the 3-4 years Healthy Beginnings booklet has information on Fundamental Movement Skills. Hopping, jumping, catching or throwing are really important for [BabyName]’s development. To help you with the steps and teach [BabyName] to gallop, check out this video  <https://www.youtube.com/watch?v=GBSTMxQMx0M> |
|  | 1268 | Try to limit using food ordering apps which have made takeaway and home delivery too easy!!! Plan healthy family meals instead:   - make a weekly grocery shopping list to include fresh or frozen fruit and vegetables - pre-chop vegetables and store them in the fridge – they are then ready to go when needed - pre-cook and freeze meals on the weekend, to be ready for your busy week ahead   Pre-cook or freeze meals are healthier and less expensive than food delivery meals. |
|  | 1271 | Aim for a rainbow plate! Tips to help [BabyName] meet the 2 ½ serves of vegetables each day:   - make funny faces with their veggies - try to put as many coloured veggies on their plate - add some veggies to their morning tea - try some vegetable muffins, frittata, chop up some vegetable sticks (steamed) and dip them in hommus   Check out this video: <https://www.youtube.com/watch?v=1u5HOURq7kQ> |
|  | 1274 | Juggling work, house chores, kids and partner? It can be a lot to manage in everyday life. Make sure you keep some time for yourself, even 5-10 minutes each day can make a difference. Set realistic expectations and seek help if you need it. For extra parenting support, see <https://raisingchildren.net.au/grown-ups/services-support/services-families/helplines> |
|  | 1277 | Drinking milk from a baby bottle can lead to tooth decay, ear infections, and may impact pre-schoolers’ growth and development. If you need support with stopping [BabyName]’s bottle use. Try:   - Encourage [BabyName]: ‘You are 3 years old now, it is time to drink from a cup’. Offer non-food rewards such as stickers to encourage positive changes. - Offer a small cup of milk after meals instead of a bottle. |
|  | 1280 | Avoid sugary drinks and foods, including soft drinks, fruit juice and flavoured milk. Drink water regularly, tap water in NSW has fluoride which is good for young teeth.  Keep your child’s teeth healthy and help them brush their teeth twice a day, and help floss at least once a day. |
|  | 1283 | It may have been tricky to limit screen time especially during the pandemic. It will benefit [BabyName] if you can keep screen time to within 1 hour per day. Here are some ideas:   - break up the 1 hour screen time into short bursts or use a timer, and turn TV off after a show ends - enjoy some active play after screen time e.g. 5 star jumps - listen to an audiobook while playing - try active screen time - dancing or sport games/virtual sports - avoid having screens in [BabyName]’s bedroom   For more information, check out <https://raisingchildren.net.au/preschoolers/play-learning/screen-time-healthy-screen-use/managing-screen-time-3-11-years> |
|  | 1286 | Praise and encourage [BabyName] when active, make time to have fun and play games you both enjoy. Active games helps with their gross and motor skills development, build confidence and is a great bonding time for you and [BabyName].  Find active games that your child likes. For example, skipping, scootering, swimming. |
|  | 1289 | Do you have a bedtime routine for [BabyName]? Aim to get [BabyName] into bed about 7-8pm to get enough hours of sleep every night. Here are some suggestions:   - have an early dinner at about 5-6pm - give them a warm bath - no screen time for least an hour before bed, instead cosy up with [Babytime] for bedtime book reading   Bedtime reading can give [BabyName] a head start in their education - it helps their listening, reading, vocabulary skills and brain development. It is a great bonding time for you and [BabyName]. |
|  | 1292 | Getting [BabyName] involved in cooking can help build their fine motor skills, measurement, language, and food knowledge. Cooking with your pre-schooler can boost their self-confidence and teaches life skills. It will help prepare them for school and success in life. Here are some tips:   - let [BabyName] choose a healthy recipe - set up a safe kitchen environment - get [BabyName] to wash the fruit and vegetables - let [BabyName] help with pouring, stirring and mixing |
|  | 1295 | Hi [FirstName], the Healthy Beginnings team will be in touch again when [BabyName] is 46 months old. You will receive a telephone support call from our clinicians and further text messages.  If you have any questions before then, send us a text and we will contact you. |

| **SMS Scheduling CHAT Study**  **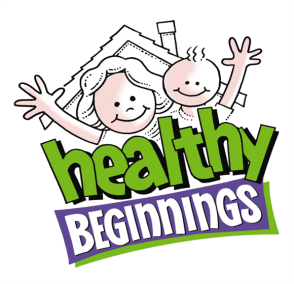**  **46-47.5 MONTHS SCHEDULE** | | |
| --- | --- | --- |
| **Reference date:**  **Date of birth** | **DAYS after ref date** | **Message content** |
|  | 1399 | Hi [FirstName], thank you for continuing participation in the Healthy Beginnings program. In the next couple of months, you will receive text messages and a telephone support call with tips on health and wellbeing for you and [BabyName]. Please text back to book in your telephone support call. |
|  | 1402 | Active families need healthy snacks. Try boiled eggs, roasted chickpeas, fruit, wholegrain crackers with nut paste or light cream cheese. Check out some easy recipes here: https://www.heartfoundation.org.au/search/recipe |
|  | 1405 | Are you ok? How are you traveling through COVID-19? There are lots of COVID-19 mental wellbeing support services that you can access. If you are struggling to cope during the pandemic, Beyond Blue has trained counsellors available 24/7. Give them a call on 1800 512 348 or check out their website <https://coronavirus.beyondblue.org.au/> |
|  | 1408 | Outdoor play has a calming effect on both you and your child. It also gives [BabyName] a space to be noisy, messy and can run free. Aim for at least one hour of energetic outdoor play per day e.g. running, jumping, climbing at playgrounds, kicking or throwing balls.  If you have concerns regarding playground or parkland restrictions during COVID-19, check https://www.planning.nsw.gov.au/Policy-and-Legislation/COVID19-response/A-guide-to-using-public-spaces-during-the-COVID19-pandemic. |
|  | 1411 | Does [BabyName] have a healthy gut? Constipation is when your child has bowel movements that are hard and dry andr less than 3 times per week. Tips:   - Eat a high fibre diet, offer your child 1serve of fruit and 2.5 serves of vegetables, and whole grain cereals daily - Drink plenty of water - Be physically active – 3 hours every day - Encourage your child to sit on the toilet 20 minutes after meals for a few minutes - Use positive words to encourage them   Check out this link if you have any concerns about your child’s bowel movement or see your GP <https://www.continence.org.au/who-it-affects/children/soiling> |
|  | 1414 | Water is the best drink for [BabyName]. Stop baby bottles, avoid sugary drinks, juices and flavoured milks. This helps protect your child’s teeth and their overall health. Ways to encourage drinking water:   - Have refillable bottles of water in the fridge - Serve water with all family meals and snacks - Put a bottle of water in your child’s lunch box - Serve iced water with orange slices and mint leaves |
|  | 1417 | Hi [FirstName], are you due for a regular health check? e.g. cervical screening, skin cancer check, blood test, blood pressure, breast check etc.  Make sure you are looking after yourself. Book in a time to see your GP. |
|  | 1420 | Your child learns through play. Playtime helps them express and process their feelings like joy, anger and fear. It is important to give [BabyName] lots of different types of play. Imaginative play, rough and tumble play helps them learn about their bodies, boosts their self-esteem, helps their social skills and motor development. |
|  | 1423 | Every minute counts! Aim for 30 minutes of exercise most days. Exercise can boost your energy level and mood, strengthen muscles, relieve stress, and improve your heart health. Tips:   - Dance with the kids - Play active ball game with the kids - Walk to day care/school/parks - Go for a walk or run at lunch time - https://www.gethealthynsw.com.au/ - For some at home workouts. See: https://www.makehealthynormal.nsw.gov.au/activity/workouts |
|  | 1426 | Aim for 7-8pm bedtime for [BabyName] so that they get enough sleep every night. Sleep is important for your child’s mood, growth, learning and development. A bedtime good routine includes:   - Dinner before 6pm - Warm bath - Aim for around 20 minutes of quiet time for cuddles, songs or story time. - No screens before bed time, try reading to your child instead - If you would like support with [BabyName] changing bedtime routine see your Child and Family Health Nurse or call Karitane 1300 227 464 or Tresillian 1300 272 736 or online chat. |
|  | 1429 | Food shopping with [BabyName] can create healthy habits and good memories for you both. To make it easier for you, try:   - Shopping early in the day to avoid crowds - Shop regularly/weekly - Don’t shop when hungry or when your child is tired - Take a shopping list- plan weekly meal plans <https://www.makehealthynormal.nsw.gov.au/food/weekly-menu-> - Avoid the aisles with snacks, biscuits, lollies, sugary drinks - Take healthy snacks and water with you |
|  | 1432 | Make dental visits part of your child’s annual health check. It is important to keep your child’s teeth healthy. Help them brush their teeth twice a day, and help floss at least once a day.  All children under 18 years can access free public dental services in NSW. Search for your oral health contact centre here  <https://www.health.nsw.gov.au/oralhealth/Pages/call-centre-search.aspx> |
|  | 1435 | Limit your child’s screen time to less than 1 hour a day. Ways to manage your child’s screen time:   - No screens at family meal times - Make bedrooms screen-free zones - Break up the 1 hour of screen time e.g. use a timer - Be a good role model yourself- https://www.makehealthynormal.nsw.gov.au/Pages/screen-time-adults.aspx |
|  | 1438 | Encourage, but don’t pressure your child to eat everything on their plate. Allow your child to feed themselves. It is best that they are in control of their own appetite, listening to their hunger and fullness cues. Put uneaten food in the fridge for another mealtime. |
|  | 1441 | Avoid sugary drinks and foods for your family. Did you know?   - 500 ml of flavoured milk can include up to 14 teaspoons of sugar - 600 ml of soft drinks can include up to 16 teaspoons of sugar - Some breakfast cereals can include up to 5 teaspoons of sugar per serve   Tap water is free, has zero sugar, quenches your thirst and includes fluoride which protects teeth. |
|  | 1444 | Just a reminder that [BabyName] will be due for their 4 year immunisation, 4 year Developmental (Bluebook) check, and the Statewide Eyesight Pre-schoolers Screening (StEPS). Please record your child’s weight and height during their developmental check. These measurements are very important for Healthy Beginnings Program. Text [BabyName]’s weight and height back to us. That would be great. |
